# Evolution of linkage and genome expansion in protocells: The origin of chromosomes

**DOI:** 10.1371/journal.pgen.1009155

**Published:** 2020-10-29

**Authors:** András Szilágyi, Viktor Péter Kovács, Eörs Szathmáry, Mauro Santos

**Affiliations:** 1 Institute of Evolution, Centre for Ecological Research, Tihany, Hungary; 2 Department of Plant Systematics, Ecology and Theoretical Biology, Eötvös Loránd University, Budapest, Hungary; 3 Center for the Conceptual Foundations of Science, Parmenides Foundation, Pullach/Munich, Germany; 4 Grup de Genòmica, Bioinformàtica i Biologia Evolutiva (GGBE), Departament de Genètica i de Microbiologia, Universitat Autonòma de Barcelona, Bellaterra, Barcelona, Spain; University of Münster, GERMANY

## Abstract

Chromosomes are likely to have assembled from unlinked genes in early evolution. Genetic linkage reduces the assortment load and intragenomic conflict in reproducing protocell models to the extent that chromosomes can go to fixation even if chromosomes suffer from a replicative disadvantage, relative to unlinked genes, proportional to their length. Here we numerically show that chromosomes spread within protocells even if recurrent deleterious mutations affecting replicating genes (as ribozymes) are considered. Dosage effect selects for optimal genomic composition within protocells that carries over to the genic composition of emerging chromosomes. Lacking an accurate segregation mechanism, protocells continue to benefit from the stochastic corrector principle (group selection of early replicators), but now at the chromosome level. A remarkable feature of this process is the appearance of multigene families (in optimal genic proportions) on chromosomes. An added benefit of chromosome formation is an increase in the selectively maintainable genome size (number of different genes), primarily due to the marked reduction of the assortment load. The establishment of chromosomes is under strong positive selection in protocells harboring unlinked genes. The error threshold of replication is raised to higher genome size by linkage due to the fact that deleterious mutations affecting protocells metabolism (hence fitness) show antagonistic (diminishing return) epistasis. This result strengthens the established benefit conferred by chromosomes on protocells allowing for the fixation of highly specific and efficient enzymes.

## Author summary

The emergence of chromosomes harboring several genes is a crucial ingredient of the major evolutionary transition from naked replicators to cells. Linkage of replicating genes reduces conflict between them and alleviates the problem of chance loss of genes upon stochastic protocell fission. The emerging organization of protocells maintaining several segregating chromosomes with balanced gene composition also allows for an increase in the number of gene types despite recurrent deleterious mutations. We suggest that this interim genomic organization enabled protocells to evolve specific and efficient enzymes and paved the way toward an accurate mechanism for chromosome segregation later in evolution.

## Introduction

No extant living organism can survive without the replication of its genetic information contained in chromosomes. Furthermore, chromosomes are a prerequisite for the evolution of complex metabolism through the appearance of specific enzymes [1]. How did chromosomes originate in the first place? The primeval self-replicating entities were probably naked RNA molecules coexisting as surface-bound populations that had to meet some stringent criteria in order to be able to evolve toward higher-level units of selection such as protocells; namely, the entities enclosing functional replicators (molecules serving as both templates and catalysts) into amphiphilic vesicles [2–4]. Protocells alleviate some obstacles faced by prebiotic systems as they increase interactions among hosted molecules and confer robustness against parasitic replicators through group selection [5–7].

However, because the genetic information within ancient protocells was likely segmented [2], unlinked replicators competed among themselves for shared resources because their relative growth rates were not under the control of the protocell. This imposed a first level of selection due to the internal competition of replicators that functioned for their own good [8]. Some offspring protocells must have inherited an unbalanced set of genes, hence be unable to grow and reproduce because of the random assortment of the genes between daughter protocells (the assortment load). Clonal selection guaranteed that those protocell lineages hosting cooperative genes would proliferate and eventually take over [5]. Although the stochastic assortment effects vanish with increasing redundancy of each sequence type, this is an unrealistic scenario for at least two reasons. First, with high redundancy there is the risk that Darwinian selection would be stopped because of dilution of favorable mutations [9]. Second, high redundancy increases the mutational load and eventually pushes the population towards extinction [10]. Furthermore, notwithstanding some claims on the putatively large number of different gene types that could be hosted by protocells [11], recent experiments have shown that the number of independent templates per protocell must be sufficiently small for protocells to be evolutionarily stable [12]. At some point in time the linkage of genes in one continuous chromosome occurred [6,13], but it is still unclear how this could have happened.

Previous attempts to explain the origin of chromosomes were limited in their scope because they modelled some very specific scenarios: only two genes, no dosage effect and absence of deleterious mutations [14], which are known to place severe limitations to the upper bound of informational length because of the error-catastrophe problem; that is, when the amount of information lost through the continuous input of deleterious mutations (mutation load) is higher than the amount of information that natural selection can recover [15,16]. The hurdles of assortment and mutation genetic loads faced by protocells should have been related problems concerning selection for linkage. Extensive theoretical work has shown that epistasis (understood as the departure from multiplicative selection) is critically important for the evolution of linkage [17,18]: when loci are subject to recurrent deleterious mutations, linkage is always favored with positive epistasis (i.e., when mutations have a weaker effect on fitness when combined). Therefore, if positive epistasis was common in early genetic systems we might expect that there was strong selection for linkage (chromosomes) because this would simultaneously reduce the two types of genetic load.

Using metabolic control theory, Szathmáry [19] showed that for a linear metabolic pathway deleterious mutations that affect different enzymes in the pathway exhibit positive epistasis when selection is for maximum flux. Starvation is a common condition in present-day bacteria [20] and probably was so in early protocells, which suggests that protocell fitness was mainly determined by the flux of a non-saturated pathway metabolizing limiting nutrients. Here we show that the major evolutionary transition “independent replicators → chromosomes” [13] was strongly favorable in early protocells and opened new routes to the evolution of complexity.

## Methods

Our goal is to understand the evolution of chromosomes and genome expansion from first principles. We assume a population of protocells containing RNA molecules (referred as ribozymes or genes) with different catalytic functions essential for the metabolism of the protocells. Besides replication these ribozymes can link together forming chromosomes, which can break and recombine. The fitness of a protocell is defined by the actual ribozyme composition, taking into account both the deviation from the optimal ribozyme composition and ribozyme dosage. The fitness-mediated group selection acting on population level together with the stochastic correction [5] defines the population dynamics. Although the stochastic corrector theory has an analytic treatment [21], this is not applicable here because of the large combinatorial number of different protocell compositions. Therefore, we use individual-based *in silico* simulations to analyze the system.

### Representation

For simplicity we assume that all RNA molecules involved in our model are 100 nucleotides long built from an alphabet of two bases, represented by 0 and 1. Catalytic activity and replicability, depend on the RNA primary structure only. (To demonstrate the possibility of an evolutionary scenario it is not necessary to link the activities to the secondary structures.) Ribozymes are organized as having a target region of *η*_*t*_ = 20 nucleotides that defines an average affinity towards the replicase (i.e., whether they are good substrates for the replicase), plus a sequence of *η*_*m*_ = 80 nucleotides that define their metabolic type and activity. A key ingredient is the suggestion [22] that primordial ribogenes were replicated in a manner similar to present-day Qβ phage RNA, with tRNA-like 3’ genome tags (i.e., a recognition site for the replicase at the end of the template; see [23]).

### Replication

For simplicity we assume that the explicitly non-represented replicase ribozymes are present in high concentration. The probability of replication of a template (*R*) depends on the number of mutated bases (*ψ*) in the target region defined by the *η*_*t*_ nucleotides as follows (see [24] for details):

$$R=1-\frac{\psi^{a}}{\beta +\psi^{a}}$$ (1)

where α and β are positive parameters. If there are no mutations in the target region then *R* = 1, and with increasing number of mutations replication probability decreases in a sigmoid way; α characterizes the steepness of the decrease and β the position of the inflexion. According to [24] we use *α* = 5 and *β* = 15. (In our model the optimal target sequence was the 20-digit long alternating sequence: 101010…) During replication mutations are introduced at a rate *μ* per nucleotide (only a single replicase acts on a template at a time).

### Metabolic activity

The metabolic activity is defined by the *η*_*m*_ long second part of the molecule. In our model we assume *D* types of metabolic activity, all essential for the survival of the protocell. A given type of metabolic activity corresponds to a specific nucleotide pattern of the metabolic region: activity type *i* is defined by the sequence of a block of eight consecutive 1s between positions 8*i*−7 and 8*i* and 0s for the rest of the sequence (*i * = * *1,2,…,*D*). In this model the maximum number of different metabolic activities is $D=\frac{\eta_{m}}{8}=10$. This choice practically excludes enzymatic promiscuity. According to [25] the activity of a gene variant *j* of activity type *i* is an exponentially decreasing function of the number of mutated nucleotides (*ψ*_*ij*_) in the metabolic region as follows:

$$A_{ij}=\frac{1}{e^{\Psi_{ij}^{2}}}$$ (2)

### Linkage

Two templates can be linked by a ligase ribozyme (present in high concentration and not represented explicitly) to form longer polymers; chromosomes *G*_*k*_+*G*_*l*_→*G*_*k*_∙*G*_*l*_, (*k*,*l* = {1,…,*D*}), where *G*_*k*_ stands for gene with metabolic activity *k* and (∙) represents the linkage and *D* is the number of essential gene types. Note that *k* and *l* are not necessarily different; i.e. the same gene can be present in multiple copies. The ligase can act in a similar manner between chromosomes $G_{k_{1}}∙G_{k_{2}}∙\ldots ∙G_{k_{M}}+G_{k_{M+1}}∙G_{k_{M+2}}∙\ldots ∙G_{k_{L}}\to G_{k_{1}}∙G_{k_{2}}∙\ldots ∙G_{k_{L}},$ (*k*_1_,*k*_2_,…,*k*_*L*_ = {1,…*D*}). We assume that the replicase travels at a constant speed along the template, which means that replication of a chromosome with *n* linked genes takes *n* times the time it takes to replicate a single gene (i.e., an *n*-fold selective disadvantage). (Note that we assume that a chromosome can be replicated by one polymerase at a time.) Regardless of whether a template is replicated or not, we assume that with probability *ν*_linkage_ two randomly chosen templates will be linked into a longer template. If the resulting chromosome is longer than a maximum limit (*MC*) there is no linkage (for computational reasons).

We also implemented recombination between two random templates with probability *ν*_recomb_. The mechanism follows a restricted copy-choice [26]: *i*) a replicase can switch from one template to another after copying a whole gene and the replicase stops after the second partner has been replicated; *ii*) two chromosomes can recombine if the gene type at the switching point is the same for both partners (e.g. ABE* *+* *DBCC* *→* *ABCC, and DBCC + ABE →* *DBE, etc.); and *iii*) if the resulting chromosome is longer than the maximum limit (*MC*), there is no recombination. The resulting template must contain at least one gene from both partners, and must be shorter than the limit *MC*. Furthermore, a chromosome can break into two parts (between genes) with probability *ν*_break_.

### Population dynamics

The population consists of *N* protocells and each protocell can host up to *S* ribozyme copies, independent of ribozyme type and activity. Protocell fitness is

$$w_{k}=\frac{D^{2}}{S\sum_{i=1}^{D}\left(\frac{1}{\sum_{j}g_{ij}Ai_{j}}\right)},(k=1,\ldots ,N)$$ (3)

where *A*_*ij*_ and *g*_*ij*_ are the metabolic activity and copy number of the *j*th variant of gene *i*. This function relies on the assumption that fitness is–as usual for microbes–essentially determined by the flux of a linear pathway of reactions catalyzed by unsaturated enzymes (c.f. Eq (5) in [27]). Eq (3) captures both the effect of mutations and enzyme dosage: it has its maximum if all enzymes have the same total activity (balanced composition) and the higher copy number increases the fitness (see Section 1 in S1 Text). The fitness function is normalized; i.e., in the optimal case when all enzymes have maximum activity (*A*_*i*_ = 1) and all *D* types present with *g*_*i*_ = *S*/*D* copy number (a protocell fulfilled with balanced composition of unmutated ribozymes) has unit value (*S* is the maximum number of genes in a protocell). If one type of activity decreases by a factor and another increases by the same factor (compensatory mutations), fitness decreases due to unbalanced composition, see Section 2 in S1 Text. (Note that if any essential gene is missing, the fitness of the protocell is zero.)

In each time step a protocell was chosen randomly according to its fitness. In this protocell one template was chosen for replication randomly according to their replication probabilities (*R*) defined by Eq (1). During replication there is a *μ* per bit mutation probability. (Recall that replication of a chromosome with *n* linked genes takes *n* times the time it takes to replicate a single gene.)

At *t*_0_ all genes have maximum metabolic and replicative activity and each cell contains a random composition of the *D* essential genes. The total number of genes in a cell is a uniform random number between 1 and *S*-1. A protocell splits into two (by hypergeometric sampling, i.e., without replacement) when the number of genes reaches the maximum size *S*. Note that the last replication will be completed even if the total number of genes at the end of replication exceeds the threshold *S*. (If replication results in at least *S* genes in total, the protocell splits.) The population dynamics follows a Moran process [28] where one daughter protocell replaces the parental protocell, and the other daughter protocell replaces a random member of the population. We terminated the simulations at *t* = 10^6^ (after 10^6^ replications). We found that during this time interval the systems always reach equilibrium. All simulations were performed in C.

## Results

### Positive (antagonistic or diminishing-returns) epistasis

As a consequence of the input of deleterious mutations in the different genes the direction of epistasis is positive, meaning that mutations have a weaker effect on protocell fitness when combined (Section 3 in S1 Text, see S1 Fig). Under this condition, decreased recombination is always favored [17,29]. It is worth mentioning that antagonistic epistasis has been predicted from studies of the effect of mutations on RNA folding [30] and analyses of RNA viruses [31], as well as in *E*. *coli* and *S*. *cerevisiae* using flux balance analysis and in silico studies of metabolic networks [32].

### Chromosomatisation and genome expansion

We first summarize the main findings and then focus on a particular scenario to understand the dynamics of the system. In all analyzed situations, chromosomes always spread despite strong within-protocell selection against them. Even if a long chromosome breaks, a diverse set of smaller chromosomes with different number of genes can be present at equilibrium. However, in all cases chromosomes with full set(s) of genes dominate the system. If the split size *S* is low (i.e., if the maximal number of genes at the time of protocell division is low), chromosomes with one full set of essential genes are present in relatively high concentration. With increasing split size the concentration of chromosomes with two (or more) full sets of genes increases; that is, we observe a genome expansion of linked genes as a function of split size. Chromosome breakage produces solitary genes and shorter chromosomes that contain no full sets of genes, reducing the average length of chromosomes and protocell fitness. Nevertheless, in the transient period chromosome breakage introduces the necessary variation to reach an optimal composition of genes in the chromosome. Without chromosome breakage the system could freeze in a suboptimal state and, in equilibrium, only a few types of chromosomes remain in the system that excludes further optimization. Finally, we have found that within-cell recombination does not affect the results qualitatively.

We now focus on a particular case assuming *D* = 3 essential genes (A, B and C). Table 1 shows the number and ratio of different types of genes in chromosomes with different gene numbers in equilibrium. The most frequent chromosome (~50%) in the population was perfectly balanced with genes ABC, and the second most frequent (~21%) chromosome with genes ABCABC. Balanced ABCABCABC chromosomes (~1.5%) were also present. In other cases the gene composition was less balanced, but on the whole there is an almost perfect equilibrium in gene composition at the population level. Breaks produce solitary genes recurrently and because of the assortment load the ratio of solitary genes of different types is not well balanced.

**Table 1:** Number of different genes in chromosomes (and their ratio relative to the total) of different lengths, sum over the whole population. Values are an average over 200,000 time steps starting at *t* = 2∙10^6^. Parameters are: *D* = 3, *S* = 21, *μ* = 10^−3^, *ν*_linkage_ = *ν*_break_ = *ν*_recomb_ = 0.01.

Number of genes in chromosomes	Frequency	Number of gene type A (ratio)	Number of gene type B (ratio)	Number of gene type C (ratio)
1	0.0617	977 (29.96%)	1010 (30.97%)	1274 (39.07%)
2	0.0796	1456 (34.63%)	1510 (35.91%)	1239 (29.46%)
3	**0.4939**	**8694 (33.32%)**	**8698 (33.33%)**	**8703 (33.35%)**
4	0.0612	1113 (34.39%)	991 (30.62%)	1132 (34.98%)
5	0.0415	798 (36.42%)	764 (34.87%)	629 (28.71%)
6	**0.2138**	**3768 (33.35%)**	**3771 (33.38%)**	**3759 (33.27%)**
7	0.0057	92 (30.77%)	109 (36.45%)	98 (32.78%)
8	0.0166	260 (29.65%)	316 (36.03%)	301 (34.32%)
9	**0.0152**	**261 (32.42%)**	**277 (34.41%)**	**267 (33.17%)**
10	0.0108	181 (31.75%)	203 (35.61%)	186 (32.63%)

**Fig 1:**
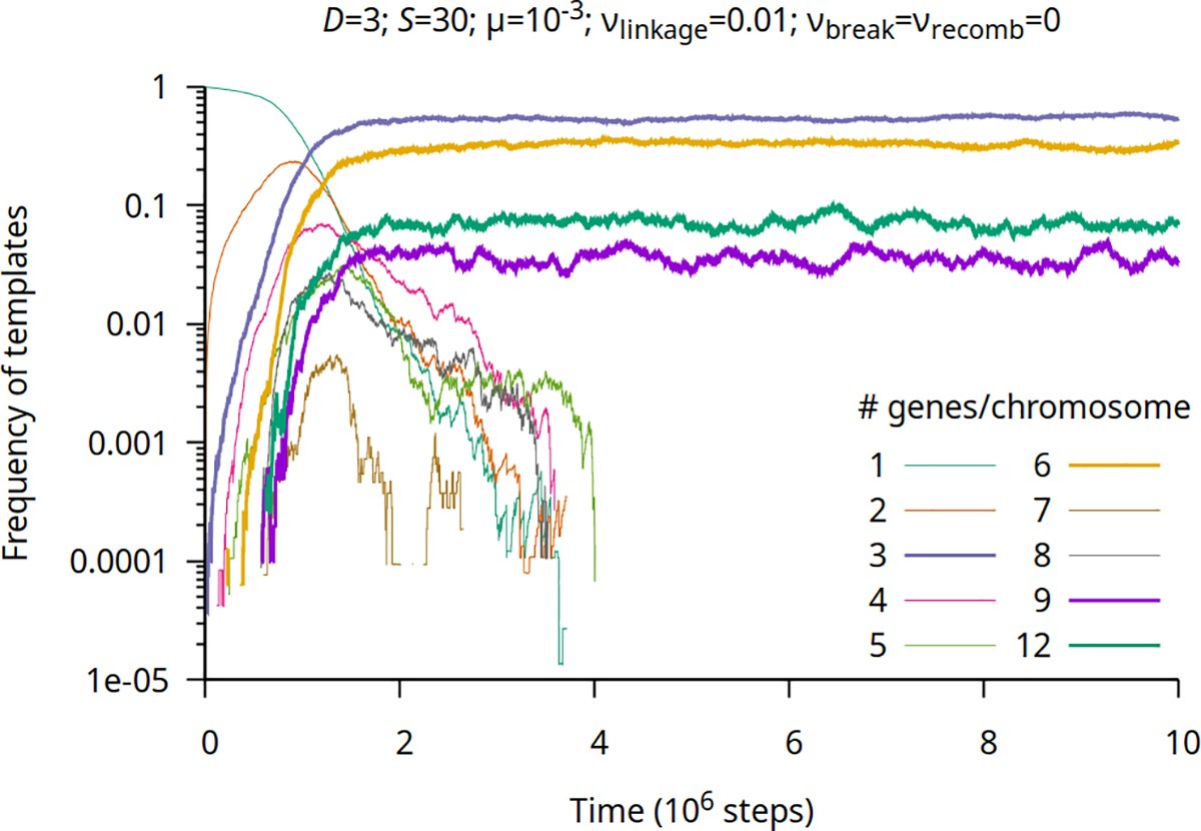
Semi-log plot of the frequency of different templates with no chromosome breakage or recombination. Frequencies are normalized on gene count (i.e., a chromosome with 3 genes counts as three when measuring the frequency), parameter values indicated at the top of the figure. Chromosomes consisting of 3∙*n* (*n* is positive integer) genes are plotted as thick lines.

Therefore, one of our main findings is that chromosomes with *n·D* sets of genes can easily arise. This likely represented an important source of novelty in protocell evolution by allowing an expanded repertoire of metabolic activities through modification of existing genes [33,34] and, at the same time, without imposing an unbearable assortment load [1]. The genome expansion of linked genes is most evident if we assume *ν*_break_ = 0, which also illustrates an important feature of the chromosomatisation dynamics (Fig 1).

Thus, because the formation of a (e.g.) 3– set balanced chromosome (ABC) has to overcome a strong within-protocell selection, what can be seen from Fig 1 is that at the beginning 2-gene chromosomes increase in frequency at the expense of solitary genes; afterwards, 3-gene (balanced) chromosomes start increasing in frequency at the expense of 2-gene chromosomes; etc. In other words, the formation of chromosomes with *n·D* sets of genes happens in a stepwise process that helps lessening the strong within-protocell selection against chromosomatisation. All imbalanced chromosomes are selected against, thus in equilibrium only chromosomes consisting of 3*n* genes are present and other gene numbers are unreachable by the system.

**Fig 2:**
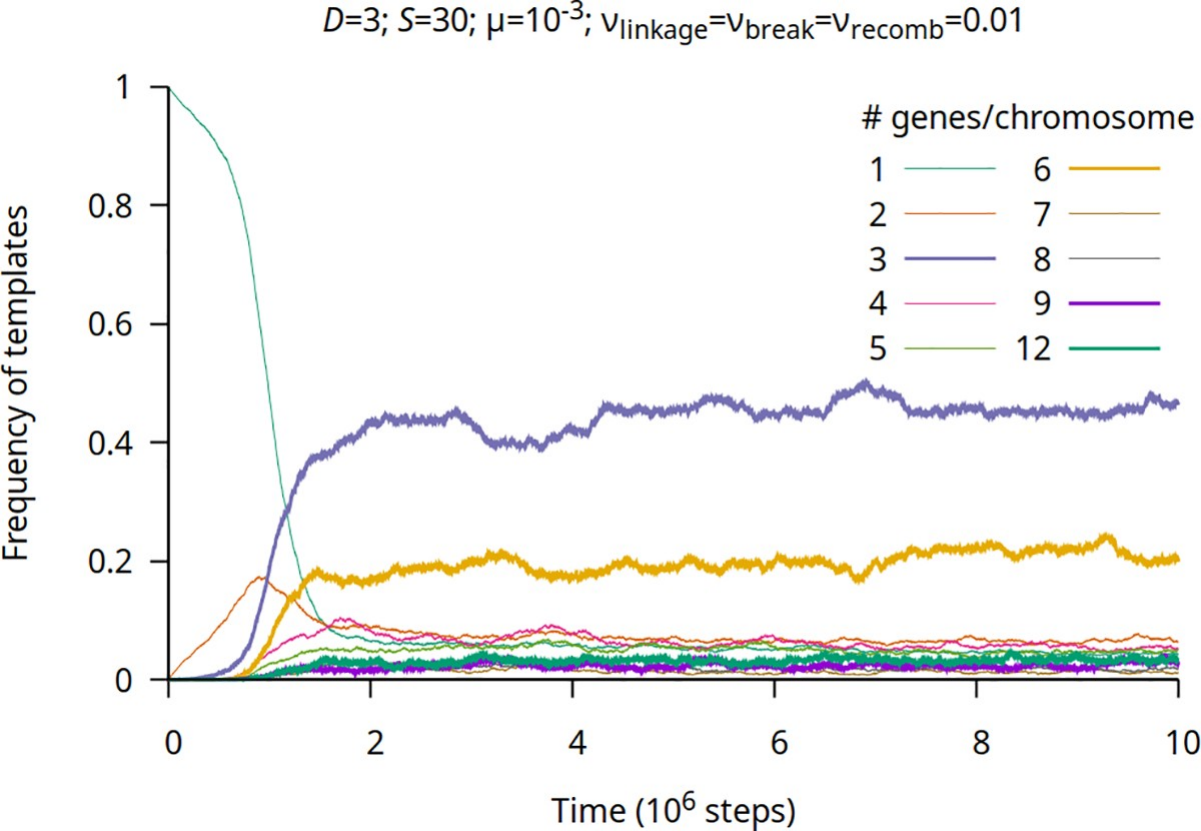
Frequency of different templates with chromosome break and recombination. Frequencies are normalized on gene count, parameter values indicated at the top of the figure (standard parameter set). Chromosomes consisting of 3·*n* (*n* positive integer) genes are plotted as thick lines. (Chromosomes with a frequency less than 1% are not shown).

By allowing for chromosome breakage (*ν*_break_ = 0.01; keeping *S* = 30), chromosomes ABC and ABCABC dominate the system (Fig 2). In this case, shorter chromosomes appear recurrently and, together with chromosomatisation, can form the basis of further adaptability of the system. Note that without breakage certain types of chromosomes are unreachable (e.g. in the case of Fig 1 2-genes, 4-genes or 8-gene chromosomes cannot be formed in equilibrium) and the system get stuck in a “frozen state”.

Both split size (*S*) and chromosome breakage (*ν*_break_>0) have important effects on the dynamics. If split size is low (*S* = 12), chromosomes with 3 genes dominate because at low split size acquisition of a chromosome with six genes is dangerous due to the early protocell fission (S2 Fig), because replication of one six-gene chromosome induces fission. As in this case the number of the chromosomes is two, in half of the cases one of the daughter compartments will be empty and inviable; this is why the system selects for three-gene chromosomes instead of six-gene chromosomes when *S* is low. With higher split size the concentration of chromosomes with six genes (mainly ABCABC type) increases (from 5% to 20%) while that of 3-gene chromosomes (mainly ABC type) decreases (from 80% to 40%), c.f. Figs 2 and S2. A further increase in split size (*S* = 50) results in decreasing concentration of chromosomes with 3 and 6 genes and increasing number of chromosomes with 9 genes (S3 Fig). High *S* results in a higher amount of longer chromosomes without a full set of genes (S3 Fig). Higher mutation rate does not alter the outcome of the chromosomatization in the sense of the ratio of different types of chromosome but increases the fluctuation in the frequencies, mainly due to the stochasticity due to the diminished number of viable protocells (S4 Fig).

The higher number of essential genes results in the domination of longer chromosomes with one (or more) full set of genes. S5 Fig shows the result of the simulation with five essential genes (*D* = 5): half of the genes are organized in chromosomes with five genes; the second most frequent is the 10 genes chromosomes class.

**Fig 3:**
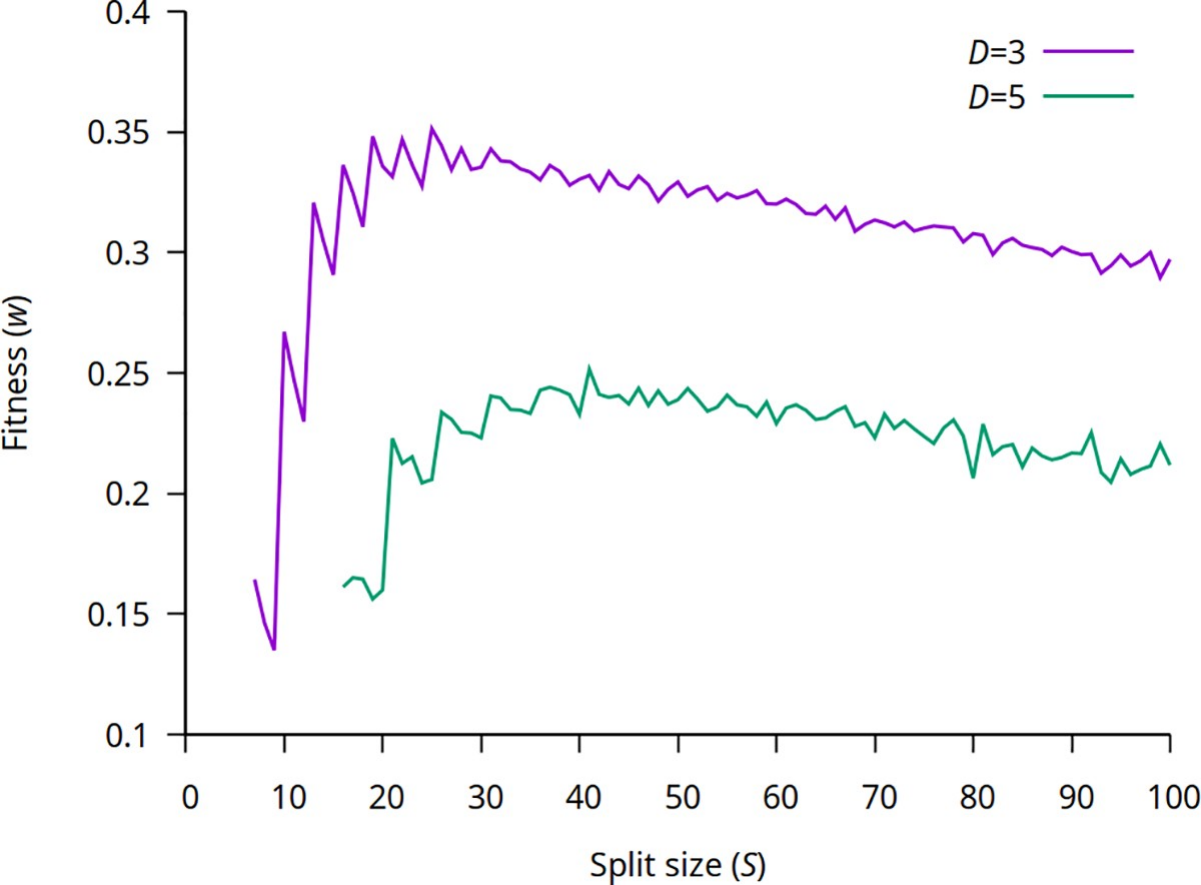
The equilibrium fitness of the population as a function of the split size (*S*) at two different numbers of essential genes *D* = 3 and *D* = 5. Average of 10 independent runs. Relevant parameters as in Fig 2 (*μ* = 10^−3^, *ν*_linkage_ = *ν*_break_ = *ν*_recomb_ = 0.01).

We have investigated the fitness and the average gene number of chromosomes as a function of the split size (*S*). As it is known from the theory of group selection [35], if the size of the group is too large the fitness tends to drop. Fig 3 clearly indicates this behavior, fitness increases with higher split size until *S*≈20 for *D* = 3 and *S*≈35 for *D* = 5, then slowly drops. The fitness curves (in the lower split size region, *S*<35) have clear peaks at S* = *n*∙*D*+1, where *n*≥3 (*n* positive integer). The explanation is clear: if *S* = *n*∙*D*, then the protocell can maintain no more than a *n*−1 balanced composition of genes, after the next replication the total number of genes reaches *S* then the protocell splits. E.g. if *D* = 3 and *S* = 12, the protocell can maintain *n* = 3 copes of ABC-type chromosomes and these chromosomes can contribute to metabolism. After the next replication the total number of genes reaches *S*, the cell splits and the “fourth” chromosome cannot help metabolism.

If, on the other hand, *S* = *n*∙*D*+1, then the protocell can maintain one more ABC-type chromosome and have higher fitness. Following the previous example, if *S* = 13 the cell does not split immediately after the appearance of the fourth ABC-type chromosome as the total gene number is under the threshold, thus four chromosomes can run metabolism and the protocell has higher average fitness. This results in peaks in the fitness curve with a period of *D*, see Fig 3. (If *D* = 3 the peaks are at 10,13,16, etc.) The decrease after these peaks is the consequence of the normalization of the fitness function: note *S* in the denominator of the fitness function in Eq (3). At higher *S* the effect of acquiring one more chromosome at *S* = *n*∙*D*+1 becomes smaller, thus the peaks diminish.

With this higher split size it is also possible to support two longer (ABCABC-type) chromosomes, which results in the same higher fitness as the four ABC-types.

With increasing split size the average gene number of chromosomes is increasing, and more or less has the same structure as the fitness curve (at lower *S* region peaks at S* = *n*∙*D*+1) (see S6 Fig for *D* = 3 and *D* = 5). With further increase in split size fitness slightly decreases as the strength of group selection weakens [35]. A detailed analysis on the effect of different parameters on the outcome can be found in Section 4 in S1 Text.

Copy-choice recombination (*ν*_recomb_) had little or no effect on the dynamics; if any, it might help the system to reach the equilibrium state faster, but there is no consistent way to measure this effect. Also note that strong selection on preserving the proper pattern of the recognition site for the replicase (c.f. Eq (3)) results in one mutated nucleotide (corresponds to *R* = 0.94) as an average–for a histogram of the number of mutations in equilibrium, see S7 Fig.

### Screening the parameter space

We performed a series of simulations to screen the parameter space to find viable region and to monitor the average number of genes in chromosomes. The relevant parameters of the model are the number of essential genes (*D*), the split size (*S*), the mutation rate (*μ*) and the linkage/break/recombination probability (*ν*), see S2 Table. The parameter values for the screen are: *D* = 2,3,…,7, *S* = 5,6,…,50, *μ* = 0, 10^−3^, 2∙10^−3^,…,8∙10^−3^. To reduce the size of the parameter space we allow chromosomatization (*ν*_linkage_ = *ν*_break_ = *ν*_recomb_ = 0.01) and exclude chromosomatization (*ν*_linkage_ = *ν*_break_ = *ν*_recomb_ = 0) as we found that intermediate values of ν make no qualitative changes in the outcome of the simulations.

**Fig 4:**
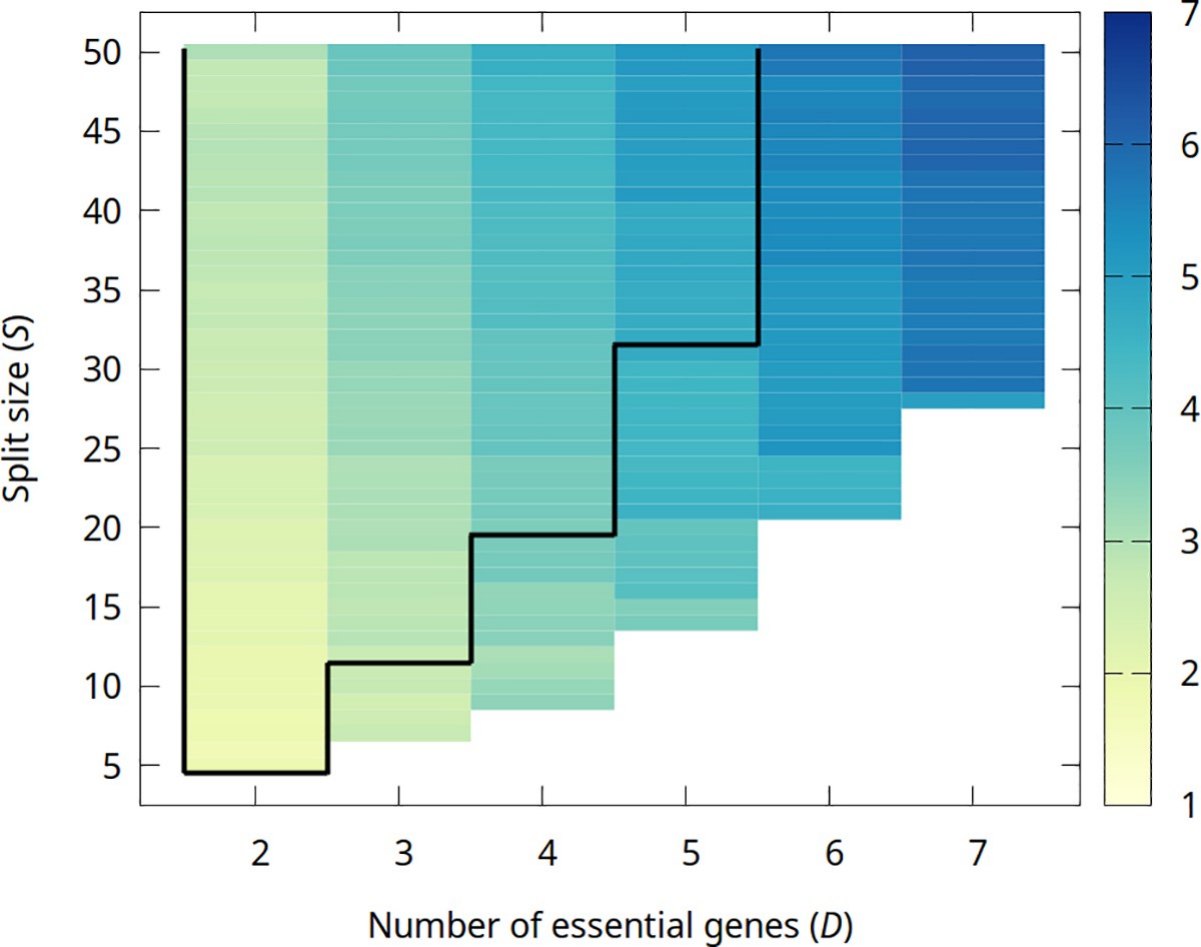
Average number of genes in chromosomes as a function of gene number (*D*) and split size (*S*) with break and recombination. Parameters are *μ * = * *10^−3^*ν*_linkage_ = *ν*_break_ = *ν*_recomb_ = 0.01. The area enclosed in black lines shows the viable region without chromosomatization.

In line with the previous results, the average length of chromosomes increases with both the number of essential genes *D* and the split size *S*. In Fig 4 one can see the average length of chromosomes (light color: solitary genes, deep blue: chromosomes with 7 genes) as a function of the number of essential genes *D* and the split size *S* assuming *μ* = 10^−3^. Chromosomatization, by decreasing the assortment load, effectively increases the sustainable number of genes: the area enclosed in black lines in Fig 4 shows the viable region without chromosomatisation. S8 Fig shows the outcome of the screening at all analyzed mutation rates. Note that the more or less periodic change in the average number of genes in chromosomes (vertical periodicity in the colors) corresponds to the balanced/unbalanced composition, cf. Figs 3 and S6. For details, see Section 5 in S1 Text.

### The effect of fast replicating parasites

We have analyzed the effect of parasitic genes; that is, genes with no metabolic contribution but replication rates higher than those of metabolic genes.

To check the evolutionary stability of the system against parasites, we changed a given amount of genes to parasites at *t* = 0. Introducing parasites at the beginning is the worst case scenario for the system due to the strong template competition within protocells, and the lack of the beneficial effect of reduced assortment load caused by chromosomes as they are not yet present.

For the whole tested parameter space, we have found that the system is robust against parasites even if their replicative advantage is unrealistically high (50% higher than that of genes with proper recognition site for replicase) and, at the beginning, 25% of all templates copies being parasitic molecules. Because of the strong effect of stochastic correction, the frequency of parasites began to decrease in the second generation (after the first replication of all protocells as an average) and basically faded away from the population after approximately 16 generations (S9 Fig). Remarkably, the stochastic correction is so strong that we have not found coexistence between metabolic genes/chromosomes and parasites in the entire investigated parameter space (Section 6 in S1 Text). The complete disappearance of parasites is due to the lack of recurrent mutations yielding new parasites.

### The effect of reduced assortment load and different intrinsic replication rates

We have analyzed the effect of two factors: i) reduced assortment load (RAL); and ii) different intrinsic replication rates (DIRR). RAL is implemented by halving solitary genes between the two daughter cells (for each *D* type), and by also halving chromosomes, independent of the metabolic activity, composition, gene number, etc. DIRR is implemented by multiplying the replication probability (defined in Eq (1)) with *B*_*i*_, a type-dependent modifier of the replication probability (*i* = 1,2,…,*D*). Small difference in intrinsic replication rates (sDIRR) are given by. *B*_1_ =1, *B*_2_ = 1,1, *B*_3_ = 1,2, etc.; while large difference (lDIRR) are given by *B*_1_ =1, *B*_2_ = 2, *B*_3_ = 3, etc. We have tested the system with the parameter combinations of Figs 2 and S2–S5.

We have found that RAL prevents the stable formation of chromosomes because linkage and breakage become almost selectively neutral. The chromosome space is populated, but no type of chromosome dominates the system (see S10 Fig). The introduction of replication imbalance has no effect on the outcome: in case of both sDIRR and lDIRR for all five tested parameter set the results are qualitatively similar to the original model: chromosomes formed and the smaller balanced compositions dominate the system. In case of combination of treatments (RAL+DIRR) there are two opposite effects: RAL acts against chromosomatization while DILL (probably) promotes it. With RAL+sDIRR chromosomes do not appear in any of the five investigated parameter sets. With RAL+lDIRR chromosomes appear after the transient period and then disappear from the system. This is probably because selection acts strongly against the assortment load induced by unlinked genes with very different replication rates and, once the within-cell disadvantage disappears because the formation of chromosomes, linkage becomes effectively neutral; see S11 and S12 Figs. For details see Section 7 in S1 Text, S1 Table summarizes the results.

## Discussion

In the “bag of genes” protocell (namely, the stochastic corrector) model [5] chromosomes must make a difference, because they decrease the assortment load (gene A is likely to find its synergistic partner gene B “in the same boat”) and alleviate intragenomic conflict (genes on the same chromosome are necessarily co-replicating [14]. This established knowledge suffered from two potential drawbacks: the unknown effects of gene dosage and the mutational load. Here we found that the gene dosage effect selects for balanced gene compositions in emerging chromosomes, and that there is a tendency for the formation of long chromosomes with “multigene families”, also with a dosage-balanced gene composition. This is a direct consequence of the protocell’s fitness function, which refers to a linear chain of enzyme-catalyzed reactions as a simplified metabolism. Therefore, our model highlights the claim that “the duplication of genetic material is rooted in the RNA world” [36].

Noteworthy is the fact that the number of sustainable gene types increases with chromosome formation. This we primarily attribute to the considerably decreased assortment load, because the latter increases with the number of gene types without linkage (for the same split size). Remarkably, unlinked genes do not beat chromosomes even for low number of gene types (*D* = 2), very high mutation rates (*μ* = 7∙10^−3^−8∙10^−3^) and high split size. In the modelled context (selection for high metabolic flux, since that ensure fast protocell growth) we find antagonistic epistasis between pairs of gene types, a fact that should also favor linkage. Furthermore, the system is remarkably resistant against parasitic mutants. The effect of the emerging multigene families combined with the dosage effect and recurrent mutations warrants detailed analysis of the mutational load that will be presented elsewhere.

A major finding is that the stochastic corrector mechanism prevails, but is shifted from gene to chromosome level. This makes sense because there is yet no accurate segregation mechanism, hence selection favors multiple chromosomes; otherwise the assortment load at the chromosome level would be prohibitive. Thus chromosomes beat genes in the simulated model, but only if the former have sufficiently high copy numbers. The dosage effect selects not only for several gene copies to be maintained, but also for chromosomes harboring balanced multigene families. This genomic composition is expected to disappear with accurate chromosome segregation and efficient transcription of genes in a later stage of evolution (awaiting further work).

Chromosome formation is a critical stage of the first major evolutionary transition [13,36]. It solidifies the protocell level of evolution (“social group maintenance” *sensu* Bourke; see [37]). It also enables the appearance of truly specific enzymes, since without linkage inefficient but multifunctional enzymes are selected for [1]. Note that here we did not model this aspect in that we assumed that enzymatic functions of genes are efficient and chemically orthogonal. A task for the future is to simulate the coevolution of enzymatic specificity/promiscuity and chromosome formation.

The above dynamical considerations are linked to some necessary change in how the genetic material was used. Before chromosomes, “RNA was more than a gene: it had a dual role harboring, genotypic and phenotypic capabilities, often in the same molecule”, and “the transition may already have begun towards the linkage of nuons to yield a composite linear RNA genome, an arrangement necessitating the origin of RNA processing” [36]. Indeed it is not difficult to build a scenario according to which chromosome formation selected for the evolution of transcription (breaking the symmetry between the two RNA strands) and the production of monocistronic RNA transcripts from chromosomes [22] not explicitly dealt with in the present model.

A further unknown is the combined effect of chromosome formation and sex between protocells. The latter is good without chromosomes when protocells with (partial) aneuploidy are more likely to fuse than healthy cells [38]. There are two potential levels of mixing, however: the reshuffling of genes and chromosomes between fusing protocells, and molecular recombination among chromosomes. Note that, the linkage-breakage dynamics realizes an ongoing indirect recombination in the model and adding an extra copy-choice recombination did not have any effect on the system. What we have detected, however, is that chromosome breakage produces shorter chromosomes and reduces protocell fitness.

We believe that the evolutionary origins of a primitive prokaryote-like genome organization will be clarified within the next few years in the context of comprehensive models integrating the discussed features. Our investigation is a further illustration how adaptation, exaptation and bookkeeping are likely to have evolved within the RNA world [39].

## Supporting information

S1 Fig The epistatic effect. The *F*(*f*,*m*) function (left panel) and the *F*(*m*) function at *f* = 2 and *f* = 4 (right panel). Parameters are: *D* = 10, *c* = 0.3, *g* = 4.
(PNG)

S2 Fig Frequency of different templates normalized on gene count with lower split size. Parameter values indicated at the top of the figure (standard parameter set, except parameter in boldface). Chromosomes consisting of 3∙*n* (*n* positive integer) genes are plotted as thick lines. (Normalization on gene count means a chromosome with 3 genes counts as three when measuring the frequency. Chromosomes with a frequency less than 2% are not shown).
(PNG)

S3 Fig Frequency of different templates normalized on gene count with higher split size. Parameter values indicated at the top of the figure (standard parameter set, except parameter in boldface). Chromosomes consisting of 3∙*n* (*n* positive integer) genes are plotted as thick lines. (For further details see S2 Fig.)
(PNG)

S4 Fig Frequency of different templates normalized on gene count with higher mutation rate. Parameter values indicated at the top of the figure (standard parameter set, except parameter in boldface). Chromosomes consisting of 3∙*n* (*n* positive integer) genes are plotted as thick lines. (For further details see S2 Fig.)
(PNG)

S5 Fig Frequency of different templates normalized on gene count with higher number of essential genes. Parameter values indicated at the top of the figure (standard parameter set, except parameter in boldface). Chromosomes consisting of 3∙*n* (*n* positive integer) genes are plotted as thick lines. (For further details see S2 Fig.)
(PNG)

S6 Fig Average gene number of chromosomes (averaged over the population) as a function of the split size (S) at two different numbers of essential genes *D* = 3 and *D* = 5. Average of 10 independent runs. Relevant parameters are as in Fig 2.
(PNG)

S7 Fig Distribution of the number of mutated nucleotides in the region defining the target affinity towards the replicase. Parameters are the same as in Fig 2. The values are averaged over 25.000 time steps starting at *t* = 10^7^. Note that, according to Eq (3) in the main text, the affinities corresponding to 0, 1, 2 and 3 mutated nucleotides are *R* = 1;0.938;0.319;0.058, respectively.
(PNG)

S8 Fig Average number of genes in chromosomes (color bar) as a function of gene number (D, x-axis) and split size (S, y-axis), with breakage and recombination at different mutation rates (from left to right and top to bottom: μ = 0,2∙10−3,3∙10−3,…,8∙10−3). Parameters are: νlinkage = νbreak = νrecomb = 0.01. The area enclosed in black lines shows the viable region without chromosomatization.
(PNG)

S9 Fig Time course of the frequency of parasites. The affinity of the parasites toward replicase is *R* = 1.5, and their frequency at *t* = 0 is 0.25. Parameters are the same as in Fig 2.
(PNG)

S10 Fig Frequency of different templates with RAL. Frequencies are normalized on gene count, parameter values indicated at the top of the figure (standard parameter set as in Fig 2) with reduced assortment load (RAL). Chromosomes consisting of 3·*n* (*n* positive integer) genes are plotted as thick lines.
(PNG)

S11 Fig The frequency of head type in chromosomes (irrespective to the length of the chromosomes) with lDIRR. Parameters are the standard parameter set as in Fig 2.
(PNG)

S12 Fig Frequency of different templates with RAL+lDIRR. Frequencies are normalized on gene count, parameter values indicated at the top of the figure (standard parameter set as in Fig 2) with reduced assortment load (RAL) and large differences in intrinsic replication rates (lDIRR). Chromosomes consisting of 3·*n* (*n* positive integer) genes are plotted as thick lines.
(PNG)

S1 Table The effect of reduced assortment load (RAL) and differential intrinsic replication rates (DIRR), see text for details. (DOCX)

S2 Table Parameters of the model. (DOCX)

S3 Table Parameters of the figures. The standard parameter set corresponds to Fig 2, the changed values marked by boldface.
(DOCX)

S1 Text Supporting text with sections on: 1) Protocell fitness is maximum if all genes (enzymes) have uniform total activity; 2) A restricted extremum behavior of the fitness function (compensatory mutations); 3) Calculation of epistasis in some simplified cases; 4) Results of different runs; 5) Screening the parameter space; 6) The effect of fast replicating parasites; 7) The effect of the reduction of assortment load and different intrinsic replication rates; 8) Parameters of the model.
(DOCX)
