# Supplementary figures and images for: Evolution of linkage and genome expansion in protocells: The origin of chromosomes
Source: PLoS Genet. 2020 Oct 29;16(10):e1009155. doi: 10.1371/journal.pgen.1009155 (PMC7665907; doi:10.1371/journal.pgen.1009155)

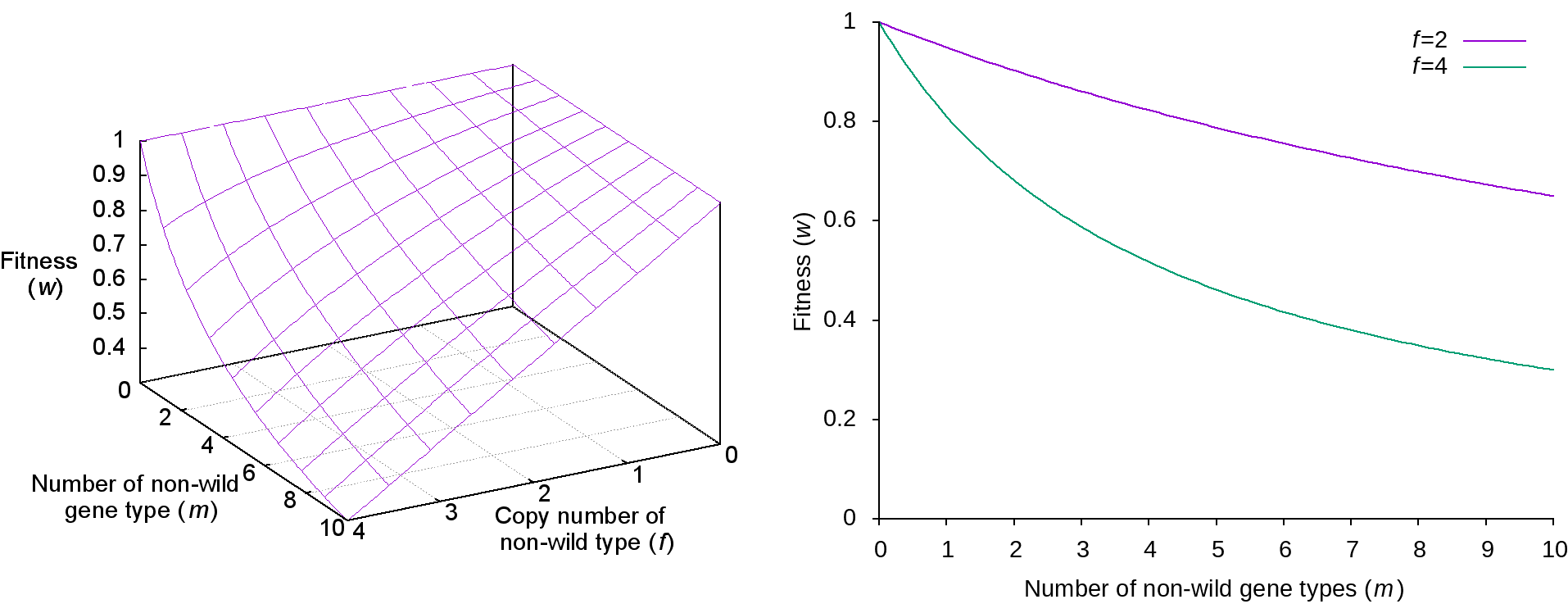

Supplement: S1 Fig — The F(f,m) function (left panel) and the F(m) function at f = 2 and f = 4 (right panel). Parameters are: D = 10, c = 0.3, g = 4. (PNG) [file pgen.1009155.s001.png]

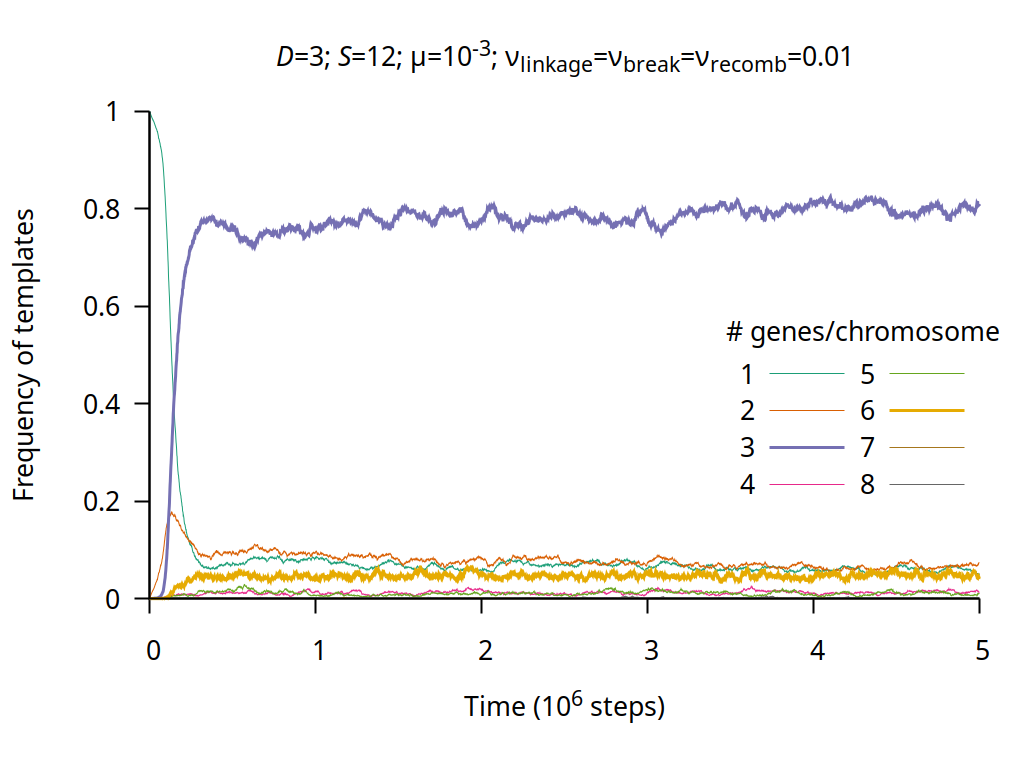

Supplement: S2 Fig — Parameter values indicated at the top of the figure (standard parameter set, except parameter in boldface). Chromosomes consisting of 3∙n (n positive integer) genes are plotted as thick lines. (Normalization on gene count means a chromosome with 3 genes counts as three when measuring the frequency. Chromosomes with a frequency less than 2% are not shown). (PNG) [file pgen.1009155.s002.png]

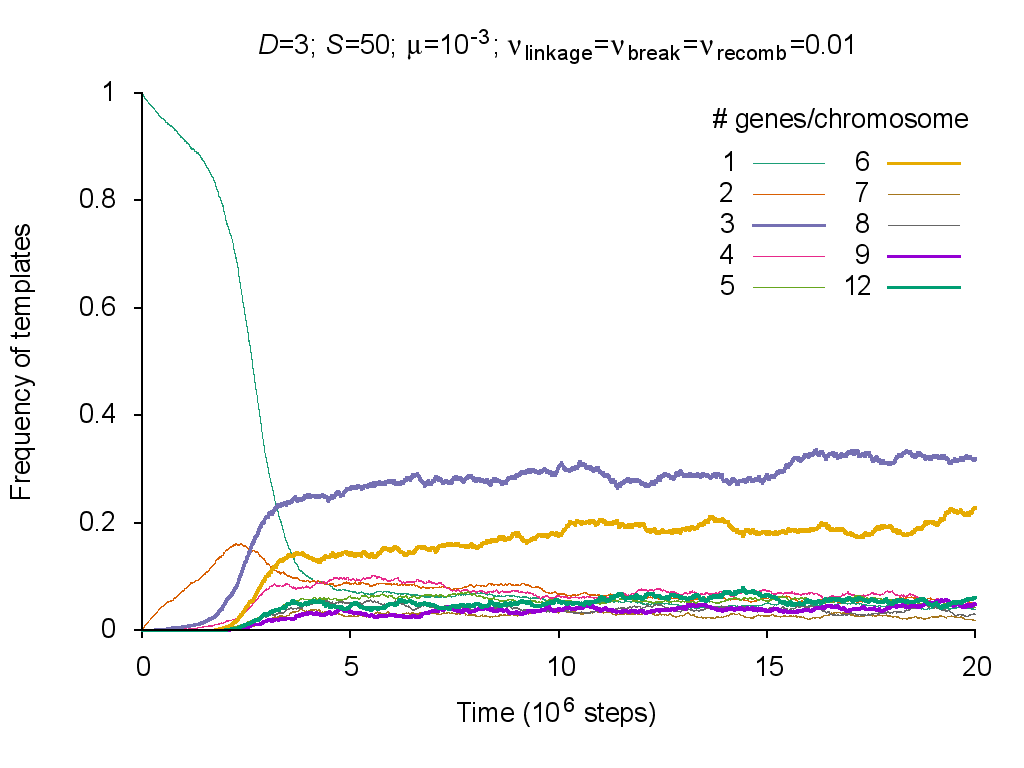

Supplement: S3 Fig — Parameter values indicated at the top of the figure (standard parameter set, except parameter in boldface). Chromosomes consisting of 3∙n (n positive integer) genes are plotted as thick lines. (For further details see S2 Fig.) (PNG) [file pgen.1009155.s003.png]

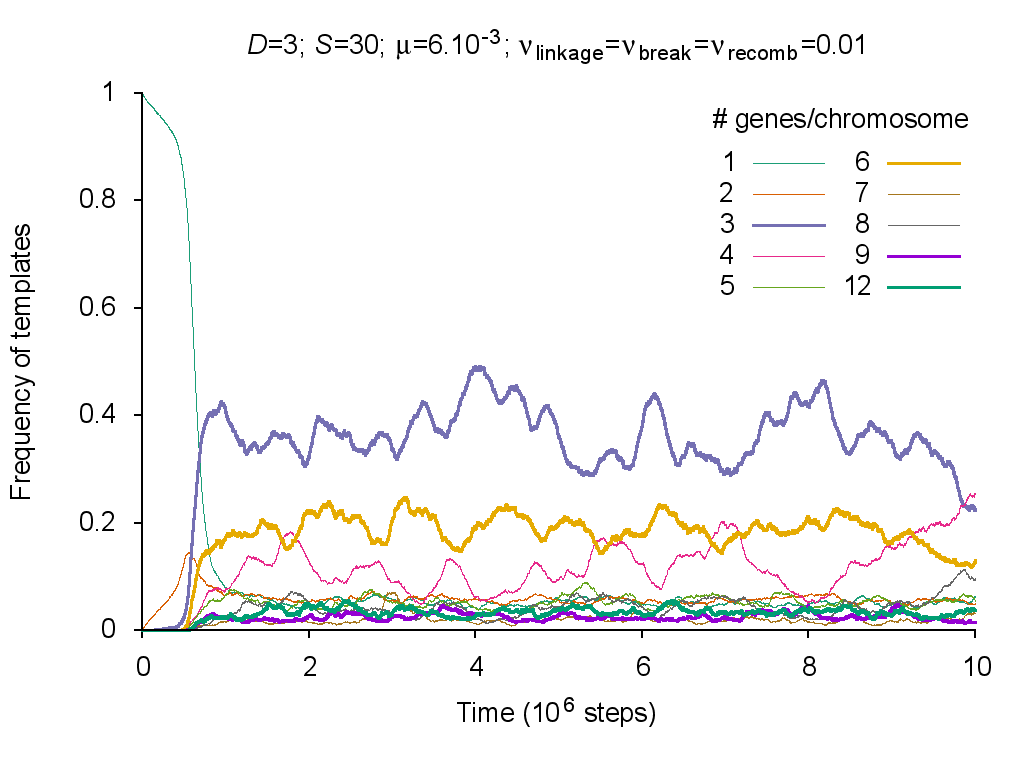

Supplement: S4 Fig — Parameter values indicated at the top of the figure (standard parameter set, except parameter in boldface). Chromosomes consisting of 3∙n (n positive integer) genes are plotted as thick lines. (For further details see S2 Fig.) (PNG) [file pgen.1009155.s004.png]

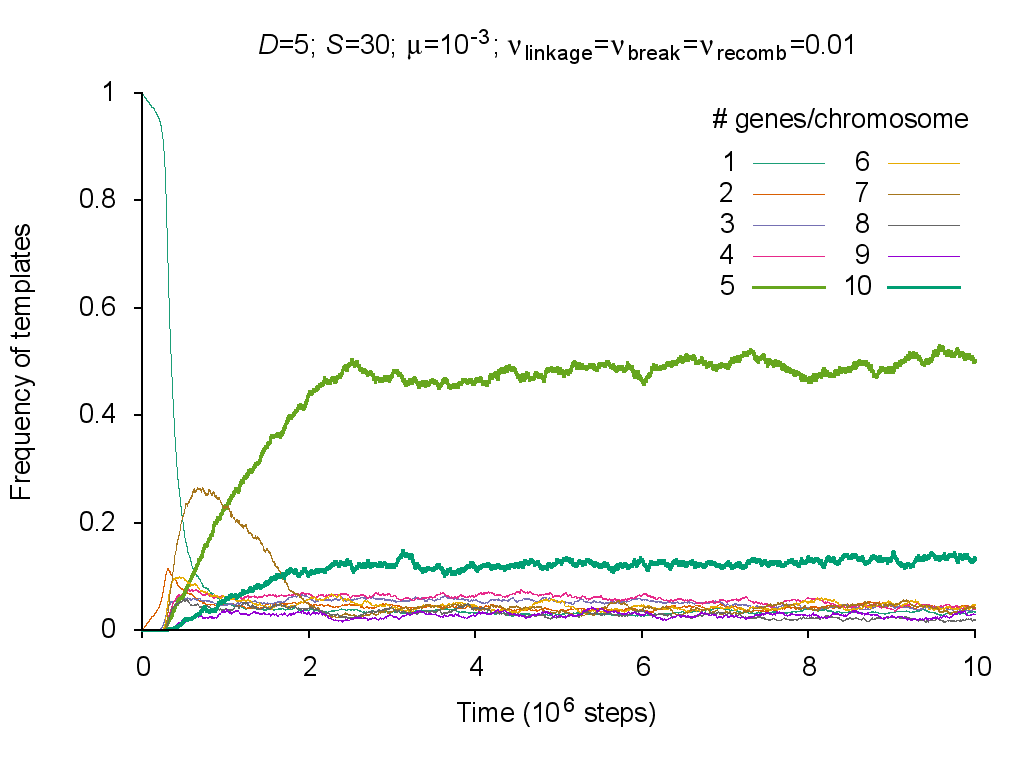

Supplement: S5 Fig — Parameter values indicated at the top of the figure (standard parameter set, except parameter in boldface). Chromosomes consisting of 3∙n (n positive integer) genes are plotted as thick lines. (For further details see S2 Fig.) (PNG) [file pgen.1009155.s005.png]

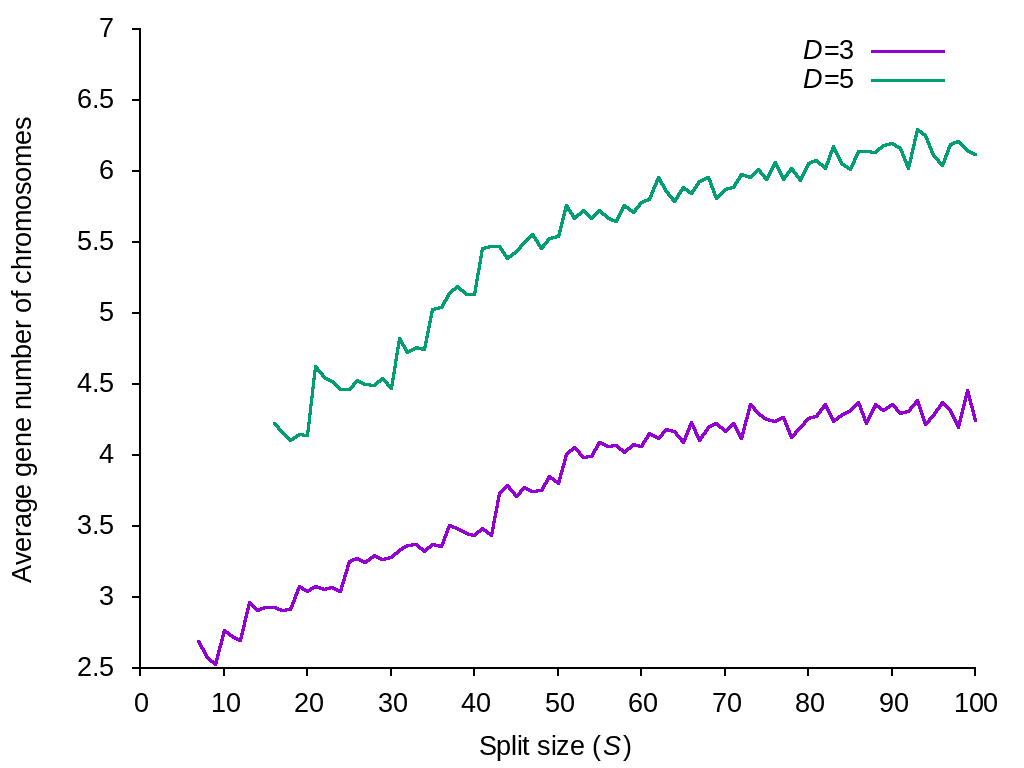

Supplement: S6 Fig — Average of 10 independent runs. Relevant parameters are as in Fig 2. (PNG) [file pgen.1009155.s006.png]

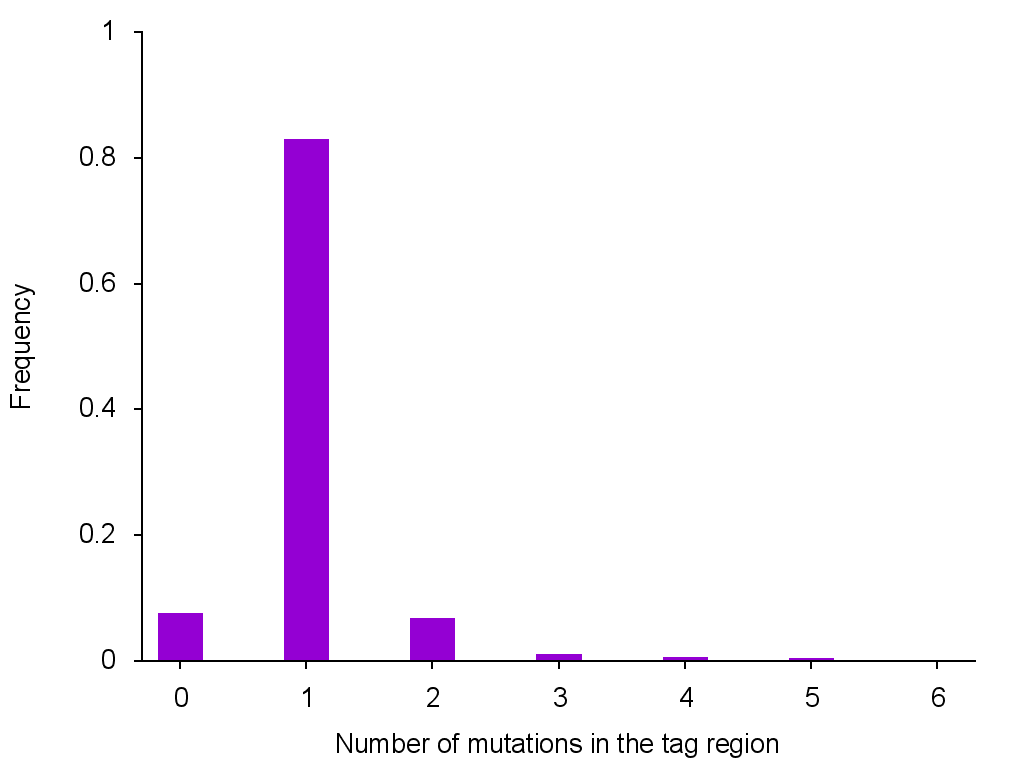

Supplement: S7 Fig — Parameters are the same as in Fig 2. The values are averaged over 25.000 time steps starting at t = 107. Note that, according to Eq (3) in the main text, the affinities corresponding to 0, 1, 2 and 3 mutated nucleotides are R = 1;0.938;0.319;0.058, respectively. (PNG) [file pgen.1009155.s007.png]

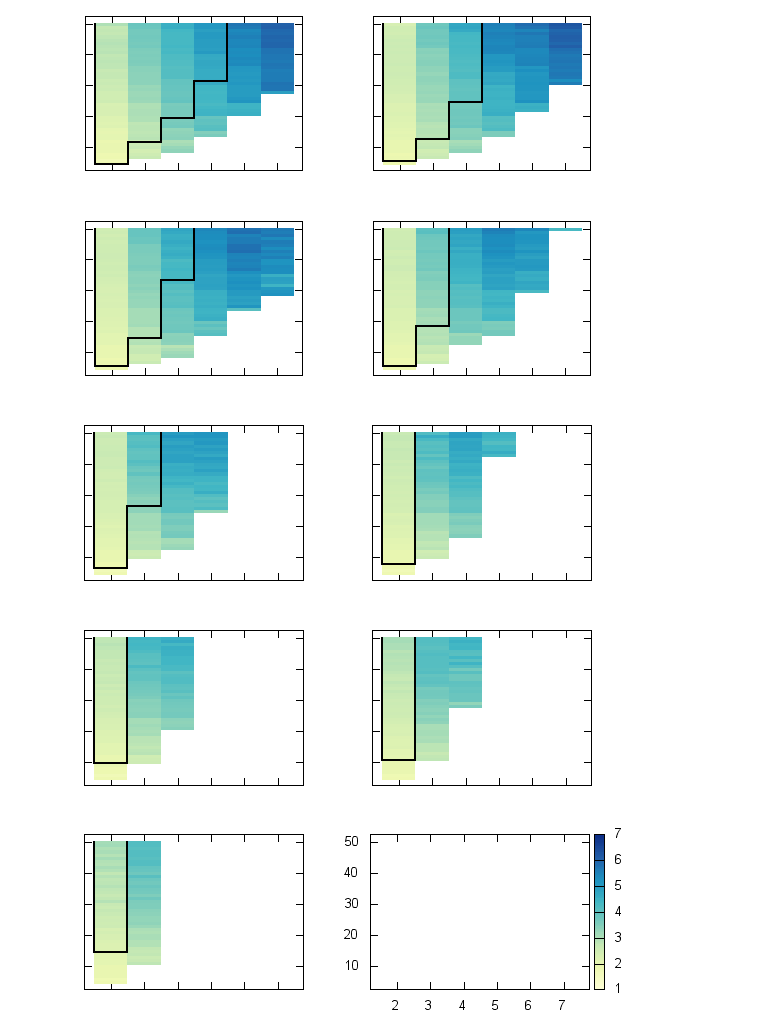

Supplement: S8 Fig — Average number of genes in chromosomes (color bar) as a function of gene number (D, x-axis) and split size (S, y-axis), with breakage and recombination at different mutation rates (from left to right and top to bottom: μ = 0,2∙10−3,3∙10−3,…,8∙10−3). Parameters are: νlinkage = νbreak = νrecomb = 0.01. The area enclosed in black lines shows the viable region without chromosomatization. (PNG) [file pgen.1009155.s008.png]

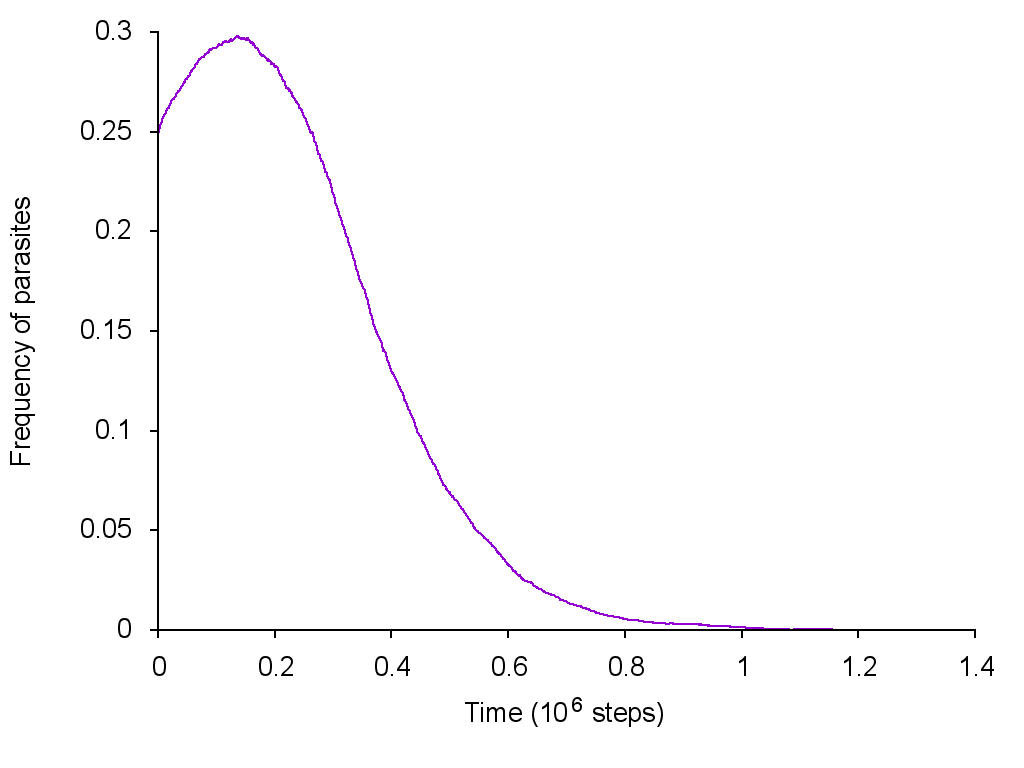

Supplement: S9 Fig — The affinity of the parasites toward replicase is R = 1.5, and their frequency at t = 0 is 0.25. Parameters are the same as in Fig 2. (PNG) [file pgen.1009155.s009.png]

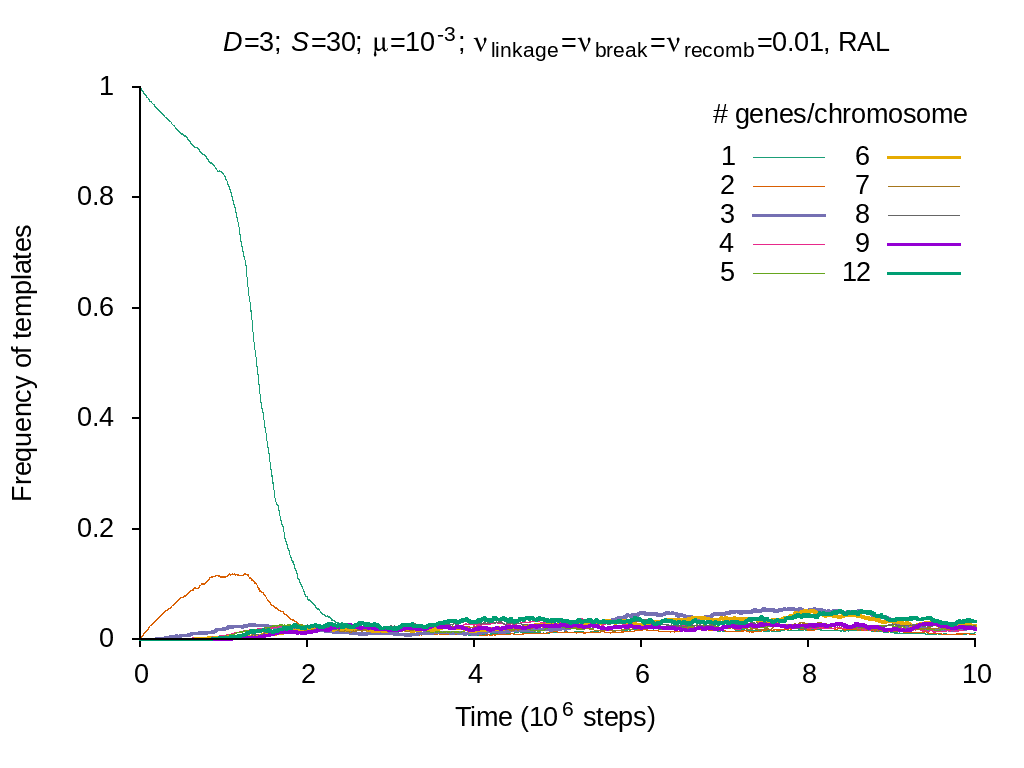

Supplement: S10 Fig — Frequencies are normalized on gene count, parameter values indicated at the top of the figure (standard parameter set as in Fig 2) with reduced assortment load (RAL). Chromosomes consisting of 3·n (n positive integer) genes are plotted as thick lines. (PNG) [file pgen.1009155.s010.png]

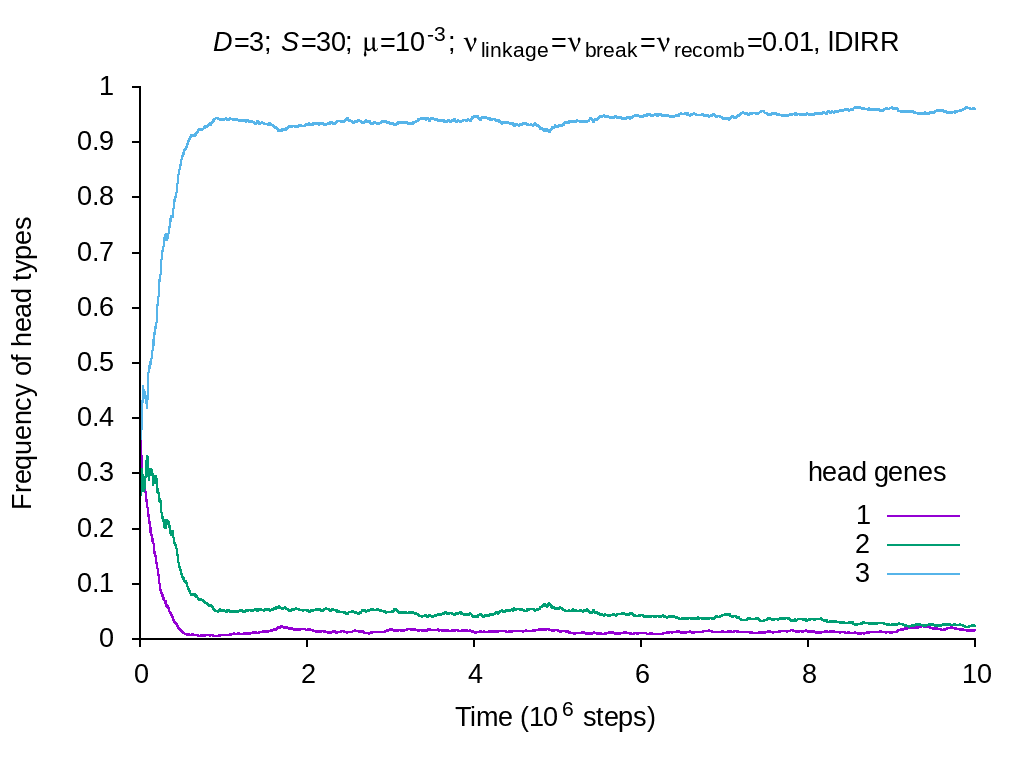

Supplement: S11 Fig — Parameters are the standard parameter set as in Fig 2. (PNG) [file pgen.1009155.s011.png]

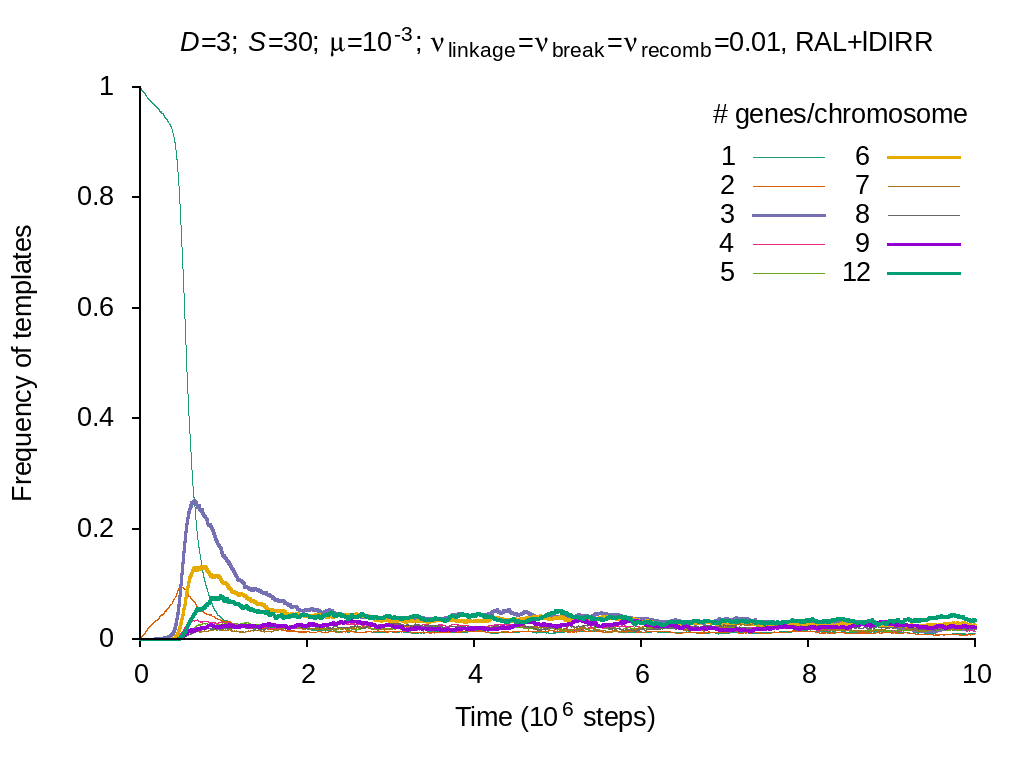

Supplement: S12 Fig — Frequencies are normalized on gene count, parameter values indicated at the top of the figure (standard parameter set as in Fig 2) with reduced assortment load (RAL) and large differences in intrinsic replication rates (lDIRR). Chromosomes consisting of 3·n (n positive integer) genes are plotted as thick lines. (PNG) [file pgen.1009155.s012.png]
