# Supplementary material for: Evolution of linkage and genome expansion in protocells: The origin of chromosomes
Source: PLoS Genet. 2020 Oct 29;16(10):e1009155. doi: 10.1371/journal.pgen.1009155 (PMC7665907; doi:10.1371/journal.pgen.1009155)
Supplement: S1 Table — (DOCX) [file pgen.1009155.s013.docx]

|  | **Fig. 2** | **Fig. S2** | **Fig. S3** | **Fig. S4** | **Fig. S5** |
| --- | --- | --- | --- | --- | --- |
| **RAL** | NOCHR | NOCHR | NOCHR | NOCHR | NOCHR |
| **sDIRR** | CHR | CHR | CHR | CHR | CHR |
| **lDIRR** | CHR | CHR | CHR | CHR | CHR |
| **RAL+sDIRR** | NOCHR | NOCHR | NOCHR | NOCHR | NOCHR |
| **RAL+lDIRR** | NOCHR^¶^ | CHR | NOCHR^¶^ | NOCHR | NOCHR^¶^ |
