# Supplementary material for: Evolution of linkage and genome expansion in protocells: The origin of chromosomes
Source: PLoS Genet. 2020 Oct 29;16(10):e1009155. doi: 10.1371/journal.pgen.1009155 (PMC7665907; doi:10.1371/journal.pgen.1009155)
Supplement: S2 Table — (DOCX) [file pgen.1009155.s014.docx]

| **parameter** | **description** | **values** | **ref.** |
| --- | --- | --- | --- |
| *D* | number of essential genes | $2-8$ | screen |
| *η_t_* | length of the target region for replicase | 20 | arbitrary |
| *η_m_* | length of metabolic region | 80 | arbitrary |
| *S* | split size | $5-50$ | screen |
| α, β | target affinity function parameters | 5,15 | [25] |
| *µ* | per bit mutation rate | 0 – 8⋅10^-3^ | screen |
| $\nu_{\mathrm{link}},\nu_{\mathrm{break}},$  $\nu_{\mathrm{recomb}}$ | probability of linkage, break and recombination | 0, 0.01 | screen |
