# Supplementary material for: Evolution of linkage and genome expansion in protocells: The origin of chromosomes
Source: PLoS Genet. 2020 Oct 29;16(10):e1009155. doi: 10.1371/journal.pgen.1009155 (PMC7665907; doi:10.1371/journal.pgen.1009155)
Supplement: S3 Table — The standard parameter set corresponds to Fig 2, the changed values marked by boldface. (DOCX) [file pgen.1009155.s015.docx]

|  | *D* | *S* | $\mu$ | $\nu$ |
| --- | --- | --- | --- | --- |
| Fig 1 | 3 | 30 | $10^{-3}$ | **0** |
| Fig 2 | 3 | 30 | $10^{-3}$ | 0.01 |
| Fig 3 | 3,**5** | 30 | $10^{-3}$ | 0.01 |
| Fig 4 | $\boldsymbol{2-7}$ | $\boldsymbol{5-50}$ | $10^{-3}$ | 0.01 |
| S2Fig | 3 | **12** | $10^{-3}$ | 0.01 |
| S3 Fig | 3 | **50** | $10^{-3}$ | 0.01 |
| S4 Fig | 3 | 30 | $\boldsymbol{6\cdot1}\mathbf{0}^{\mathbf{-3}}$ | 0.01 |
| S5 Fig | **5** | 30 | $10^{-3}$ | 0.01 |
| S6 Fig | 3,**5** | 30 | $10^{-3}$ | 0.01 |
| S8 Fig | $\boldsymbol{2-7}$ | $\boldsymbol{5-50}$ | $\boldsymbol{0-8\cdot}\boldsymbol{1}\boldsymbol{0}^{\boldsymbol{-3}}$ | 0.01 |
| S9 Fig | 3 | 30 | $10^{-3}$ | 0.01 |
