# Supplementary material for: Evolution of linkage and genome expansion in protocells: The origin of chromosomes
Source: PLoS Genet. 2020 Oct 29;16(10):e1009155. doi: 10.1371/journal.pgen.1009155 (PMC7665907; doi:10.1371/journal.pgen.1009155)
Supplement: S1 Text — Supporting text with sections on: 1) Protocell fitness is maximum if all genes (enzymes) have uniform total activity; 2) A restricted extremum behavior of the fitness function (compensatory mutations); 3) Calculation of epistasis in some simplified cases; 4) Results of different runs; 5) Screening the parameter space; 6) The effect of fast replicating parasites; 7) The effect of the reduction of assortment load and different intrinsic replication rates; 8) Parameters of the model. (DOCX) [file pgen.1009155.s016.docx]

**SUPPORTING INFORMATION**

**Evolution of linkage and genome expansion of protocells:**

**the origin of chromosomes**

András Szilágyi, Viktor Péter Kovács, Eörs Szathmáry, and Mauro Santos

C**ontents**

|  |  |  |
| --- | --- | --- |
| 1. Protocell fitness is maximum if all genes (enzymes) have uniform total activity | 2 |  |
| 1. A restricted extremum behavior of the fitness function (compensatory mutations) | 3 |  |
| 1. Calculation of epistasis in some simplified cases | 4 |  |
| 1. Results of different runs | 5 |  |
| 1. Screening the parameter space | 9 |  |
| 1. The effect of fast replicating parasites | 11 |  |
| 1. The effect of the reduction of assortment load and different intrinsic replication rates | 12 |  |
| 1. Parameters of the model | 15 |  |
|  |  |  |
|  |  | |

1. **Protocell fitness is maximum if all genes (enzymes) have uniform total activity**

For simplicity we will use the unnormalized fitness function of Eq. (3) in the main text

|  | $w=\frac{1}{\sum_{i=1}^{D} \left( \frac{1}{\sum_{j} g_{ij}A_{ij}} \right)}$ | (S1) |
| --- | --- | --- |

and would like to find its maximum. The problem can be simplified by finding the maximum of

|  | $w=\frac{1}{\sum_{i=1}^{D} \frac{1}{e_{i}}}$ | (S2) |
| --- | --- | --- |

where $e_{i}=\sum_{j} g_{ij}A_{ij}$, a combined measure of copy number and activity (the total, weighted activity of enzyme type *i* ). The maximum is subject to constant total activity $\sum_{i} e_{i}=C$.

As a first step we introduce the Lagrange function

|  | $L\left( e_{1},e_{2},\ldots,e_{D} \right)=\frac{1}{\sum_{i=1}^{D} \frac{1}{e_{i}}}+\lambda\left( \sum_{i=1}^{D} \frac{1}{e_{i}}-C \right)$ | (S3) |
| --- | --- | --- |

or in compact form

|  | $L\left( \mathbf{e} \right)=F\left( \mathbf{e} \right)+ \lambda\left( \sum_{i=1}^{D} \frac{1}{e_{i}}-C \right)$ | (S4) |
| --- | --- | --- |

where $\mathbf{e}=(e_{1},e_{2},\ldots,e_{D})$ is the vector of activities.

In a maximum subjected to the total activity constraint $\frac{\partial L(\mathbf{e})}{\partial e_{i}}=0$ (for all *i*). After simple calculations we arrive at

|  | $\frac{\partial L(\mathbf{e})}{\partial e_{i}}=\frac{F^{2}(\mathbf{e})}{e_{i}^{2}}+\lambda.$ | (S5) |
| --- | --- | --- |

From this, in maximum _­_$\hat{e}_{i}=F\left( \mathbf{e} \right)\sqrt{-\frac{1}{\lambda}}$ . Substituting back into the total activity constraint, after simple rearrangement we get $\lambda=-\frac{D^{2}F^{2}(\mathbf{e})}{C^{2}}$. Combining this with the condition $\frac{\partial L(\mathbf{e})}{\partial e_{i}}=0$ we arrive at $\hat{e}_{i}=\frac{C}{D}$, which means that the protocell fitness has its maximum if all enzymes have the same total activity.

1. **A restricted extremum behavior of the fitness function (compensatory mutations)**

Let us assume here that both enzyme activity (*A*) and copy number (*g*) are the same for all types of genes. By introducing $e=Ag$ and using the unnormalized fitness function as above

|  | $F=\frac{1}{\sum_{i=1}^{D} \left( \frac{1}{\sum_{j} g_{ij}A_{ij}} \right)}=\frac{1}{\sum_{i=1}^{D} \frac{1}{e}}=\frac{e}{D}$ | (S6) |
| --- | --- | --- |

We will prove that if for one gene the *e* value (the combination of activity and copy number) reduces by an amount $d(<i)$ and, as a compensatory effect, for one other type increases by *d*, the metabolic flux will decrease.

The calculation of the unaltered flux can also be done in the following way

|  | $F=\frac{1}{\sum_{i=1}^{D} \frac{1}{e}}=\frac{1}{{\left( \begin{matrix} D \\ D-1 \end{matrix} \right)\prod_{i=1}^{D-1} e}/{\prod_{i=1}^{D} e}}=\frac{\prod_{i=1}^{D} e}{D\prod_{i=1}^{D-1} e}=\frac{e}{D}.$ | (S7) |
| --- | --- | --- |

By introducing $e\to e+d$ and $e\to e-d$ for any two gene types, the modified fitness can be expressed as

|  | $F^{'}=\frac{\left( e+d \right)\left( e-d \right)e^{D-2}}{\left( D-2 \right)\left( e+d \right)\left( e-d \right)e^{D-3}+\left( e+d \right)e^{D-2}+\left( e-d \right)e^{D-2}}.$ | (S8) |
| --- | --- | --- |

After some simple manipulation we get the following result

|  | $F^{'}=\frac{e}{\left( D+2\frac{d^{2}}{e^{2}+d^{2}} \right)}<F,$ | 4 (S9) |
| --- | --- | --- |

which indicates that fitness reduces even if the mutations are compensatory.

1. **Calculation of epistasis in some simplified cases**

*Two genes*. Let us denote the two unmutated gene (enzyme) types by *A* and *B*, and when they carry a deleterious mutation by $A'$ and $B'$. The activity of each unmutated enzyme is 1, and that of the mutated enzyme is $c(<1)$. We assume that both enzymes are present in one copy. From Eq. (1) in the main text, the fitness of different combinations can be easily calculated

|  | $w_{AB}=1, w_{A^{'}B}=w_{AB^{'}}=2\frac{c}{c+1}, w_{A^{'}B^{'}}=c$ | (S10) |
| --- | --- | --- |

where we assume $D=S=2$ to get normalized fitness.

Let us define epistasis ($\varepsilon$) as the fitness decrease of the double mutant divided by twice the fitness decrease of a single mutation

|  | $\varepsilon=\frac{1-c}{2\left( 1-2\frac{c}{c+1} \right)}=\frac{1+c}{2}<1$ | (S11) |
| --- | --- | --- |

i.e., the epistasis is positive. If we assume that both genes are present in *g* each copies, a similar calculation gives the same result (in this case $S=2g$).

*More genes.* Let us assume that there are *D* different gene types. The activity of the wild type and the mutant are 1 and *c*, respectively. Let *m* denote the number of different gene types for which at least one copy is mutant, thus $D-m$ gene types lack mutation. We assume *g* number of copies for each types of gene. For a mutated gene type *f* copies bear a single mutation, and $g-f$ copies are wild type. With these assumptions the fitness can be calculated as ($S=Dg$)

|  | $F=\frac{D\left[ g-f\left( 1-c \right) \right]}{D\left[ g-f\left( 1-c \right) \right]+mf\left( 1-c \right)}$ | (S12) |
| --- | --- | --- |

The fitness as a function of *m* and *f* is clearly nonlinear, indicating epistatic effects. The strength of the epistasis depends on parameter values. $g-f(1-c)$ is always positive as $f\leq g$ and $0<c<1$, thus the $F(m)$ function is always convex downward, clearly indicating positive epistasis (on both additive and multiplicative scales; c.f. S1 Fig).

| 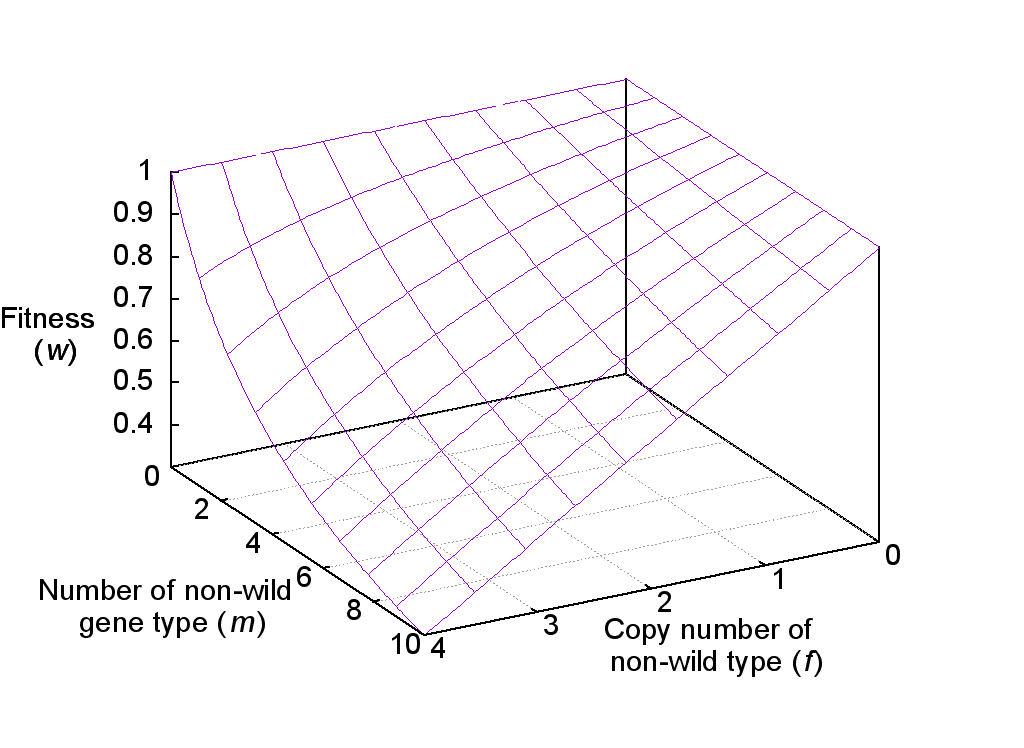 | 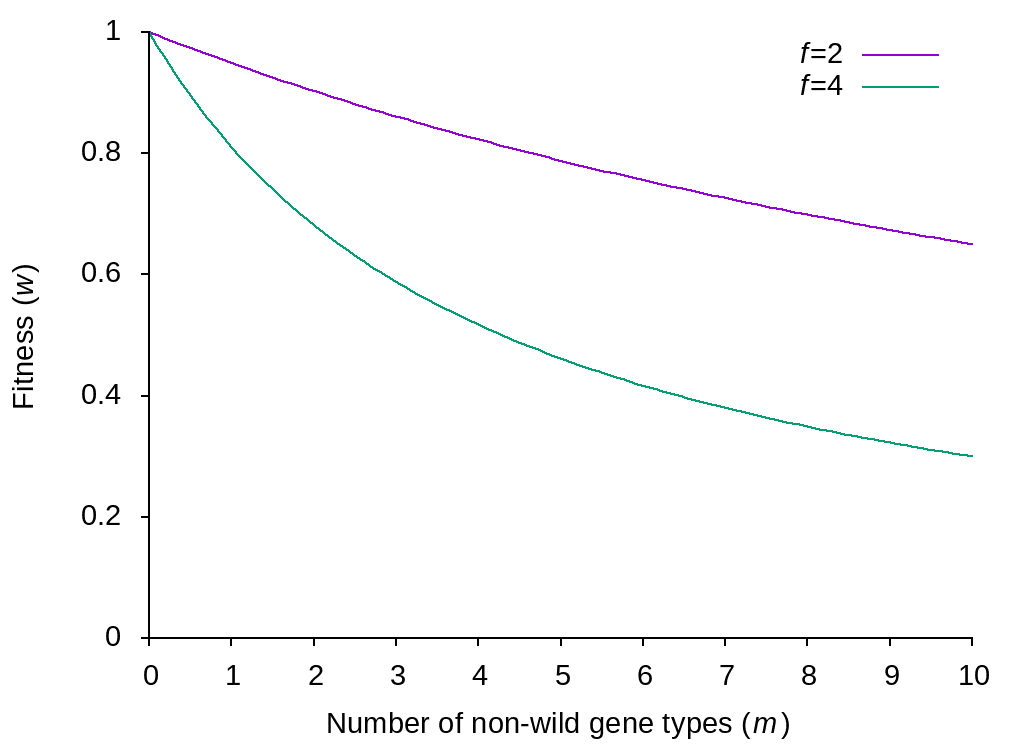 |
| --- | --- |

**S1 Fig. The epistatic effect.** The $F(f,m)$ function (left panel) and the $F(m)$ function at $f=2$ and $f=4$ (right panel). Parameters are: $D=10, c=0.3, g=4$.

1. **Results of different runs**

We plot here a number of graphs varying different parameters as indicated in the figures. We compare these simulations to the “reference run” of Fig 2 ($D=3$, $S=30$, $\mu={10}^{-3}$, $\nu_{\mathrm{linkage}}=\nu_{\mathrm{break}}=\nu_{\mathrm{recomb}}=0.01$). In the following, we will indicate the changed parameters only. S2 Fig shows the effect of smaller split size ($S=12$). The result clearly indicates that smaller split size reduces the amount of larger chromosomes, because acquisition of a chromosome with six genes (ABCABC-type) is dangerous due to the early protocell fission.


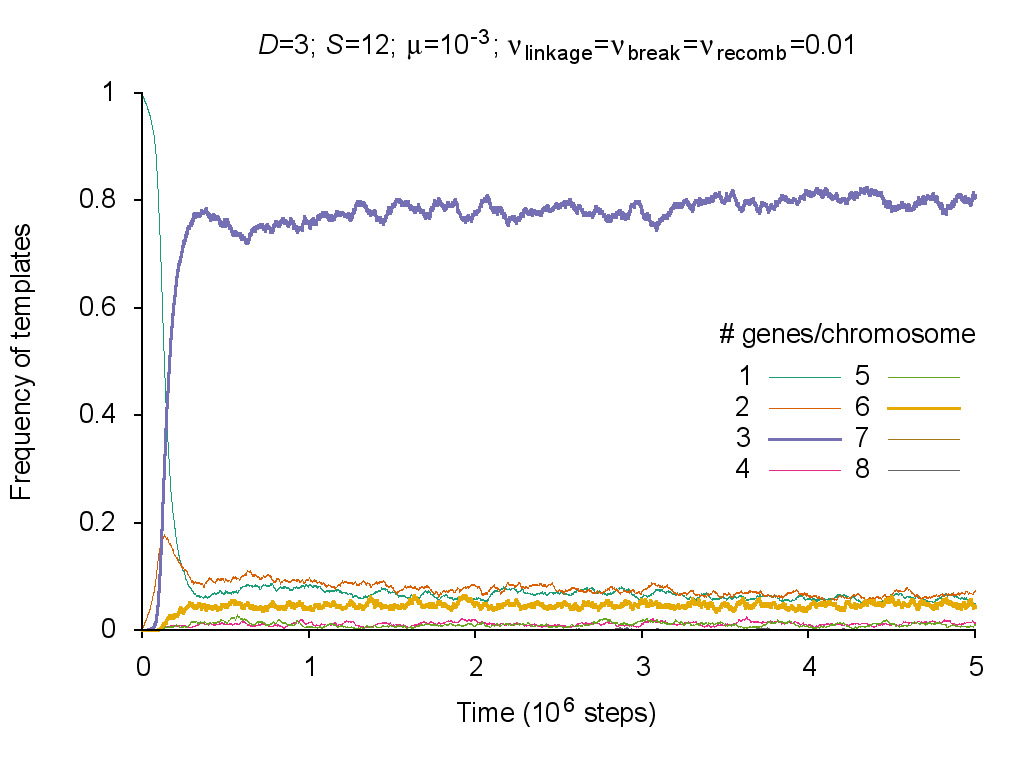


**S2 Fig.** **Frequency of different templates normalized on gene count** **with lower split size**. Parameter values indicated at the top of the figure (standard parameter set, except parameter in boldface). Chromosomes consisting of $3\cdot n$ (*n* positive integer) genes are plotted as thick lines. (Normalization on gene count means a chromosome with 3 genes counts as three when measuring the frequency. Chromosomes with a frequency less than 2% are not shown).

A simulation presented in S3 Fig shows the opposite situation; the effect of larger split size $\left( S=50 \right)$. This split size allows a higher concentration of chromosomes with 9 genes, as these chromosomes cannot cause too early division. Parallel with the increasing concentration of 9-genes chromosomes, the frequency of 3-genes chromosomes reduces while the frequency of 6-genes chromosomes remains the same. Higher split size results in a higher total amount of no $n\cdot D$ type chromosomes that cannot have one or more full set of essential genes.


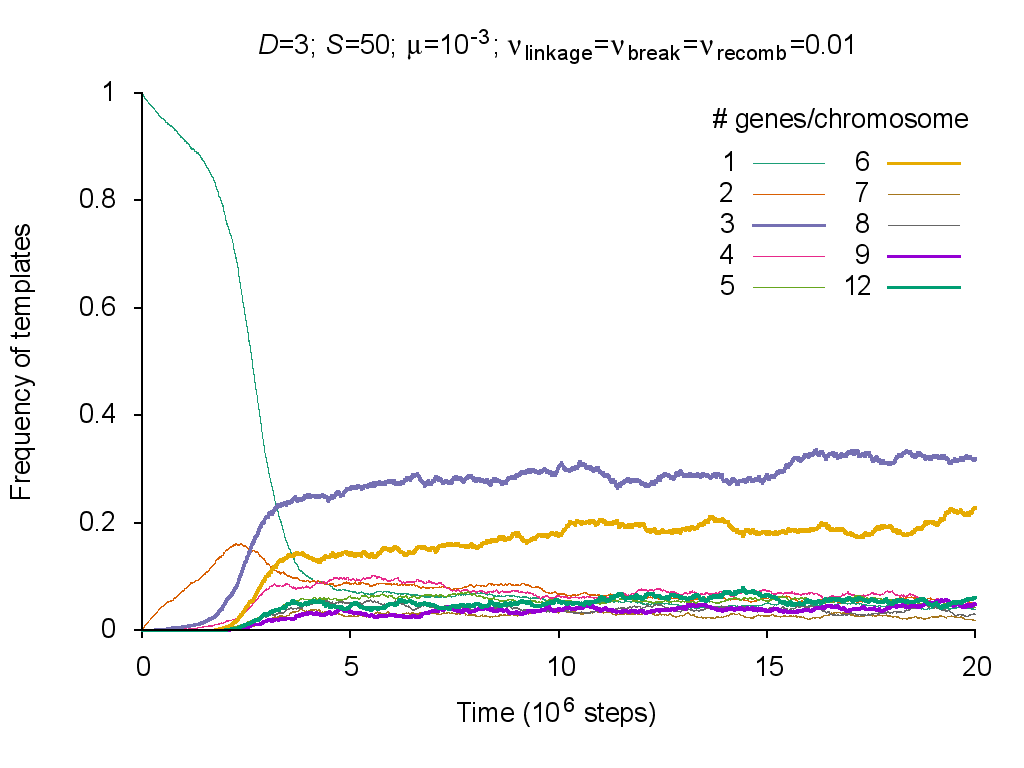


**S3 Fig.** **Frequency of different templates normalized on gene count** **with higher split size**. Parameter values indicated at the top of the figure (standard parameter set, except parameter in boldface). Chromosomes consisting of $3\cdot n$ (*n* positive integer) genes are plotted as thick lines. (For further details see S2 Fig.)

We have also investigated the effect of higher mutation rate (S4 Fig). The qualitative behaviour does not change and the ratio of different types of chromosomes remained mainly unaltered (cf. Fig 2). The fluctuation in the frequency is mainly due to the stochasticity generated by the lower concentration of viable protocells (approximately 35% of the protocells have nonzero fitness, and the mean fitness is about 0.02).

If the number of essential genes is higher longer chromosomes appear. The dominant chromosome class consists of one or more full set of genes (they are of types). S5 Fig shows the result of a simulation with $D=5$, where the 5 genes chromosomes (almost all are ABCDE-type) dominate the system and the second most populated class is the class of 10 genes chromosomes. At $S=30$, the 15-genes chromosomes cannot appear as these chromosomes cause immediate protocell fission.


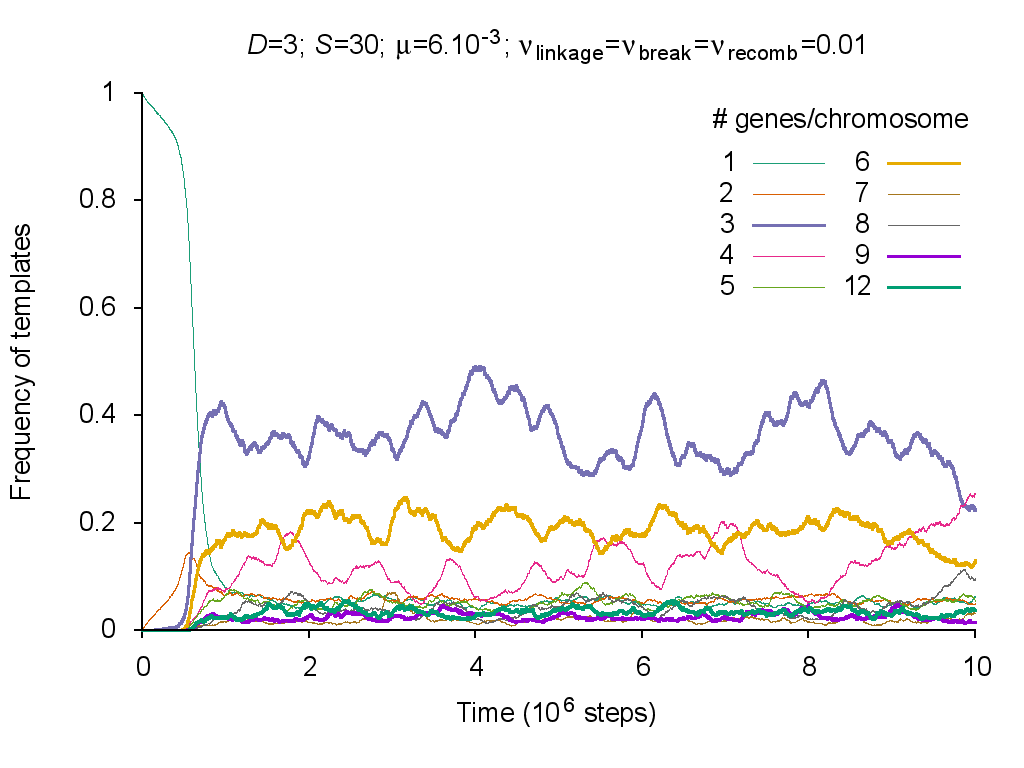


**S4 Fig.** **Frequency of different templates normalized on gene count** **with higher mutation rate**. Parameter values indicated at the top of the figure (standard parameter set, except parameter in boldface). Chromosomes consisting of $3\cdot n$ (*n* positive integer) genes are plotted as thick lines. (For further details see S2 Fig.)


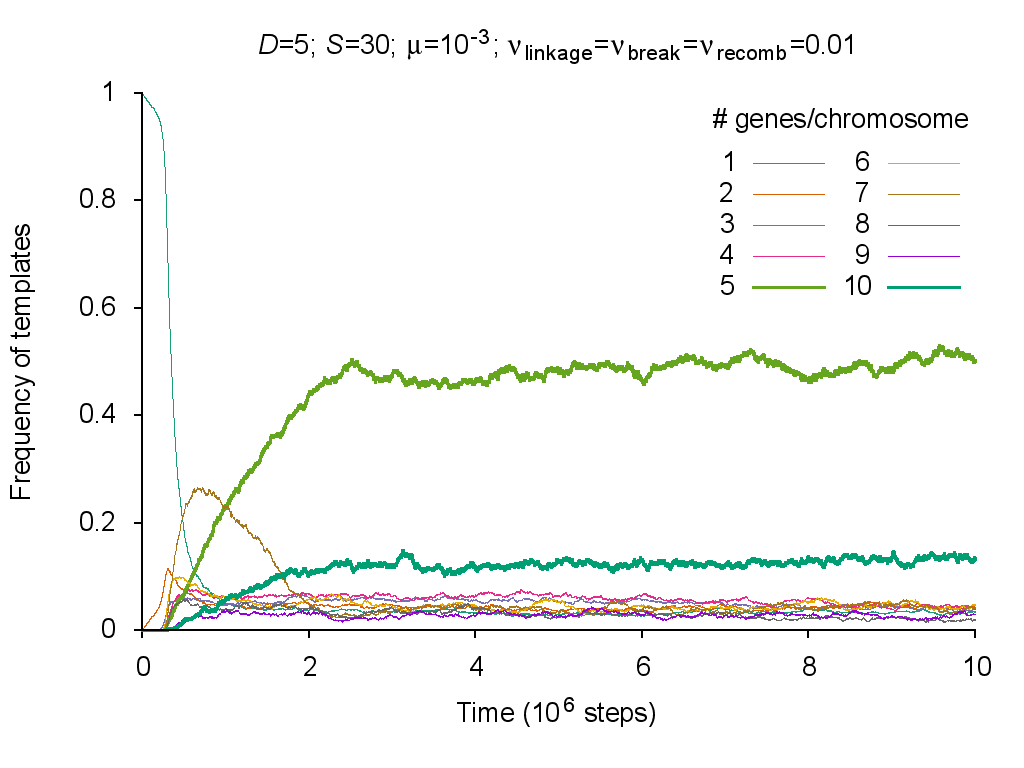


**S5 Fig.** **Frequency of different templates normalized on gene count** **with higher number of essential genes**. Parameter values indicated at the top of the figure (standard parameter set, except parameter in boldface). Chromosomes consisting of $3\cdot n$ (*n* positive integer) genes are plotted as thick lines. (For further details see S2 Fig.)

The course of the average gene number of chromosomes as a function of the split size (S6 Fig) has a saturating characteristic. In the $10<S<60$ region of split size the gene number increases with the split size in a linear way, indicating the dosage effect: more balanced composition of genes results in higher fitness. This effect diminishes if $S\gg D$; cf, the $S>70$ region in S6 Fig.

| 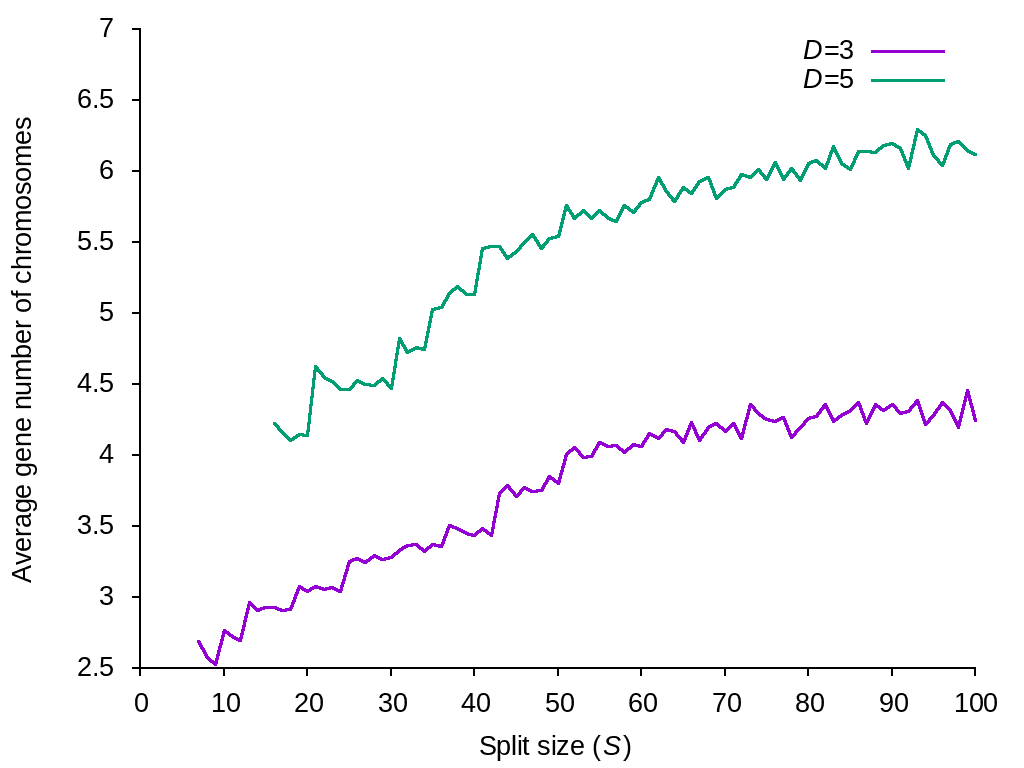 |
| --- |
| **S6 Fig.** **Average gene number of chromosomes (averaged over the population) as a function of the split size (*S*) at two different numbers of essential genes** $\boldsymbol{D=3}$ **and** $\boldsymbol{D=5}$**.** Average of 10 independent runs. Relevant parameters are as in Fig 2. |

S7 Fig shows the distribution of the number of mutations in the region defining the target affinity towards the replicase in the evolved population (at $t={10}^{7}$). The peak at one mutated nucleotides corresponds to $R=0.938$ replication probability, cf. Eq. (3) in the main text. As the replication probability *R* is a fast decreasing function of the number of mutated nucleotides $\psi$, the replication probability of genes/chromosomes with more than four errors in the relevant region is negligible.

| 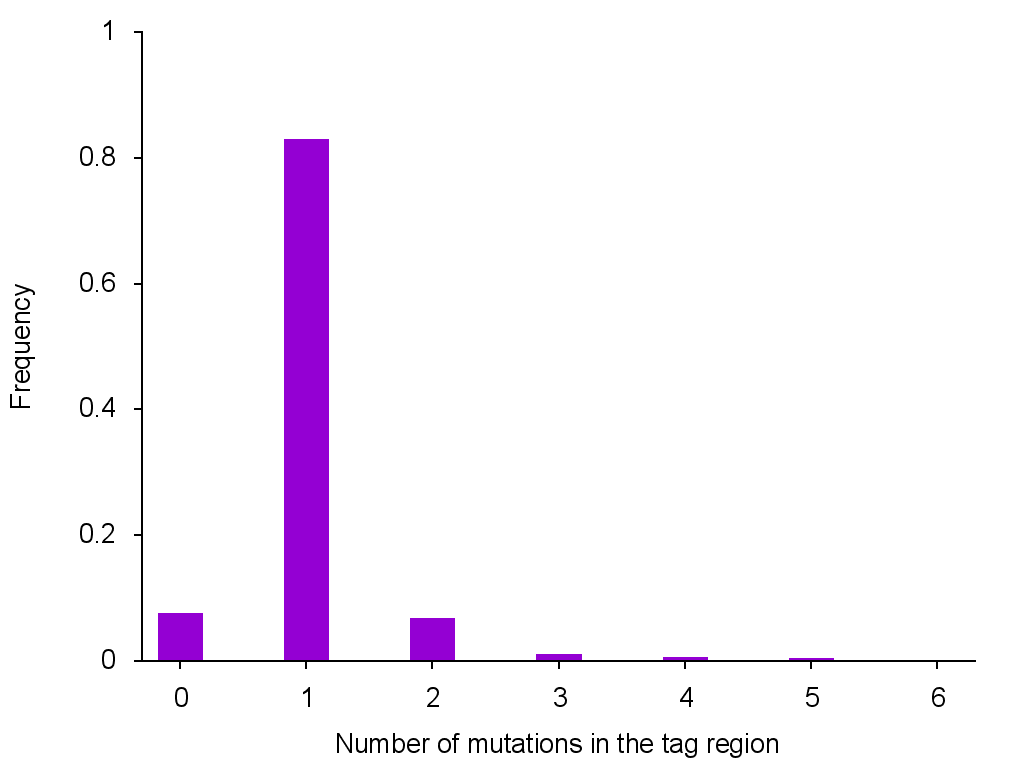 |
| --- |
| **S7 Fig.** **Distribution of the number of mutated nucleotides in the region defining the target affinity towards the replicase**. Parameters are the same as in Fig 2. The values are averaged over 25.000 time steps starting at $t={10}^{7}$. Note that, according to Eq. (3) in the main text, the affinities corresponding to 0, 1, 2 and 3 mutated nucleotides are $R=1;0.938;0.319;0.058$, respectively. |

1. **Screening the parameter space**

We have also analyzed the average gene number of chromosomes in the parameter space spanned by the split size (*S*), the number of essential genes (*D*), and the mutation rate (μ). Different panels of S8 Fig correspond to different mutation rates from $\mu=0$ to $\mu=8\cdot{10}^{-3}$ (increasing mutation rates from left to right and from top to bottom). Each plot shows the average gene number (with color coding) as an average over 200,000 time steps starting at at $t=2\cdot{10}^{6}$. The periodic pattern of the average number of genes at fixed *D* is in agreement with the observation of the special role of $S^{*}=n\cdot D+1$ split size, at which split density the average gene number increases as one more full set of genes can be harbored (see Results and Discussion in the main text). The non-coherent fine structure at higher *D* (and higher *S*) regime is mainly due to the stochastic effects. It can be seen that the viable region in the *S*-*D* planes shrinks: with the increasing mutation rate the system can maintain fewer types of genes.

The effect of chromosomatization on the sustainable amount of information (the number of sustainable essential genes, *D*) has also been investigated. We ran simulations with no chromosomatization $\left( \nu_{\mathrm{linkage}}=\nu_{\mathrm{break}}=\nu_{\mathrm{recomb}}=0 \right)$ and the viable region of the parameter space enclosed in black lines. The possibility of chromosome formation increased the number of sustainable genes by a factor of 2 to 3.


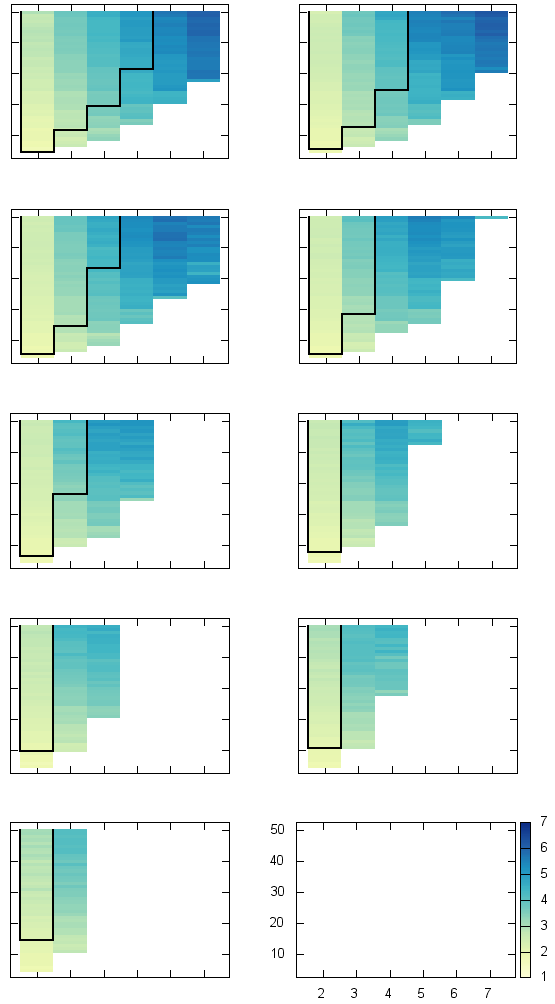


**S8 Fig. Average number of genes in chromosomes (color bar) as a function of gene number (*D*, *x-*axis) and split size (*S*, *y*-axis)**, with breakage and recombination at different mutation rates (from left to right and top to bottom: $\mu=0, 2\cdot{10}^{-3},3\cdot{10}^{-3},\ldots,8\cdot{10}^{-3}$). Parameters are:$\nu_{\mathrm{linkage}}=\nu_{\mathrm{break}}=\nu_{\mathrm{recomb}}=0.01$. The area enclosed in black lines shows the viable region without chromosomatization.

1. **The effect of fast replicating parasites**

We have analyzed the effect of parasites; that is, genes with higher *R* affinity towards the replicase and without metabolic activity. We have made a series of runs over the relevant part of parameter space $\left(D\text{, }S\text{, }\mu\text{, }\nu\right)$ with different replicase affinities *R* and different concentrations of parasites. In order to analyze the worst case scenario for the system, we made two assumptions: i) we add parasites at $t=0$ and, thus, parasites compete mainly with singe genes to ignore the reduced assortment load caused by chromosomes; and ii) we assumed that mutations do not act on the region that defines parasites’ activity towards replicase (i.e., constant affinity towards the replicase). Linkage between parasites was ignored. We have found that in the entire part of the parameter space used in the simulations, the parasites disappear from the system. Remarkably, stochastic correction is so strong that we have not found coexistence between metabolic genes/chromosomes and parasites.

S9 Fig shows the time course of the frequency of parasites with unrealistically high replication rate $R=1.5$ (50% higher than the maximum for non-parasites) and a very high initial concentration (25% of the genes are parasites). As one can see, the stochastic correction eliminates parasites relatively fast. The characteristic time of a generation (the time necessary for division of all vesicles at an average) can be estimated as $=\frac{S}{2}N$ , where *S* is the split size and *N* is the number of protocells in the population. With the parameters of S9 Fig $\tau=75,000$; exclusion of the parasite starts during the second generation and finishes around the 16^th^ generation.

1. **The effect of the reduction of assortment load and different intrinsic replication rates**

We have analyzed the effect of two factors: i) reduced assortment load (RAL); and ii) different intrinsic replication rates (DIRR). RAL is implemented as follows. When the number of genes in a compartment reaches the maximum size *S* the split follows the following rules: solitary genes are halved between the two daughter cells (for each *D* type), chromosomes are also halved, independent of the metabolic activity, composition, gene number, etc. If the copy number of a solitary gene or chromosome type is odd then the last member is assorted randomly. These simple changes reduce the assortment load efficiently.

| 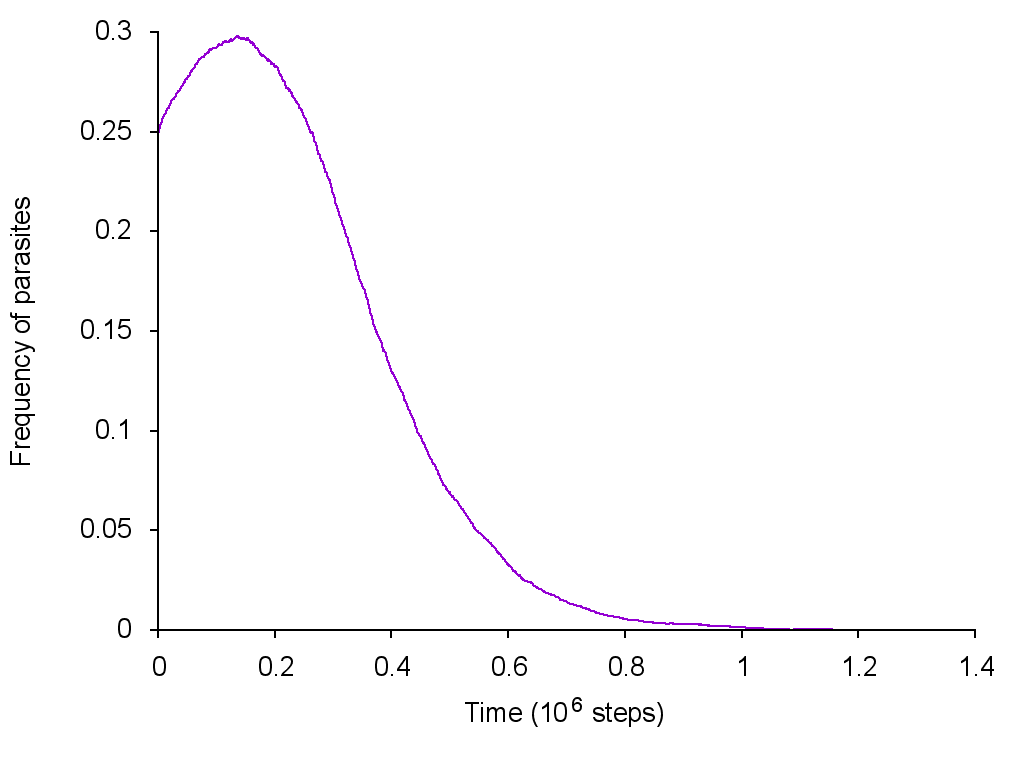 |
| --- |
| **S9 Fig.** **Time course of the frequency of parasites.** The affinity of the parasites toward replicase is $R=1.5$, and their frequency at *t* = 0 is 0.25. Parameters are the same as in Fig 2.  DIRR is implemented by modifying Eq. (1) as follows:  $R^{'}=B_{i}\left( 1-\frac{\psi^{\alpha}}{\beta+\psi^{\alpha}} \right)=B_{i}\cdot R,$  where $B_{i}$ is a type-dependent modifier of the replication probability ($i=1,2,\ldots,D$). We have tested two cases: small difference (sDIRR) and large difference (lDIRR). In the former case $B_{i}=1+0.1\cdot(i-1)$ (i.e. $B_{1}=1, B_{2}=1.1, B_{3}=1.2$, etc.); while in the latter case $B_{i}=i$ (i.e. $B_{1}=1, B_{2}=2, B_{3}=3$, etc.).  We have tested the two treatments independently and in combination, using the parameters in Figs. 2, S2, S3, S4 and S5, see also Table S2. These choices cover the relevant versions of the system. Results are summarized in Table S1.   \|  \| **Fig. 2** \| **Fig. S2** \| **Fig. S3** \| **Fig. S4** \| **Fig. S5** \| \| --- \| --- \| --- \| --- \| --- \| --- \| \| **RAL** \| NOCHR \| NOCHR \| NOCHR \| NOCHR \| NOCHR \| \| **sDIRR** \| CHR \| CHR \| CHR \| CHR \| CHR \| \| **lDIRR** \| CHR \| CHR \| CHR \| CHR \| CHR \| \| **RAL+sDIRR** \| NOCHR \| NOCHR \| NOCHR \| NOCHR \| NOCHR \| \| **RAL+lDIRR** \| NOCHR^¶^ \| CHR \| NOCHR^¶^ \| NOCHR \| NOCHR^¶^ \|   **S1 Table. The effect of reduced assortment load (RAL) and differential intrinsic replication rates (DIRR, see text for details).** The parameter sets are denoted by the number of corresponding abbreviations. NOCHR means no chromosomization, CHR means chromosomatization (with the same characteristics as in the original model), NOCHR^¶^ means a temporal appearance of chromosomes in the transient phase.  As one can see RAL prevents the formation of chromosomes, in contrast to the original model. In case of RAL linkage and breakage are almost selectively neutral, the chromosome space is populated, but no type of chromosome dominates the system. We have found the same result in all the five investigated parameter sets. The result obtained with the parameters of Fig. 2 can be seen in S10 Fig.  The introduction of replication imbalance has no effect on the outcome: in case of both sDIRR and lDIRR for all five tested parameter set the results are qualitatively similar to the original model: chromosomes formed and the smaller balanced compositions dominate the system. As the result of the large replicative advantage in case of lDIRR in the head of the chromosomes there are the genes with largest *B*. The frequency of different types of gene in the heads of the chromosomes (parameters of Fig. 2) can be seen in S11 Fig. As one can see gene type 3 (largest *B*) dominates the heads.  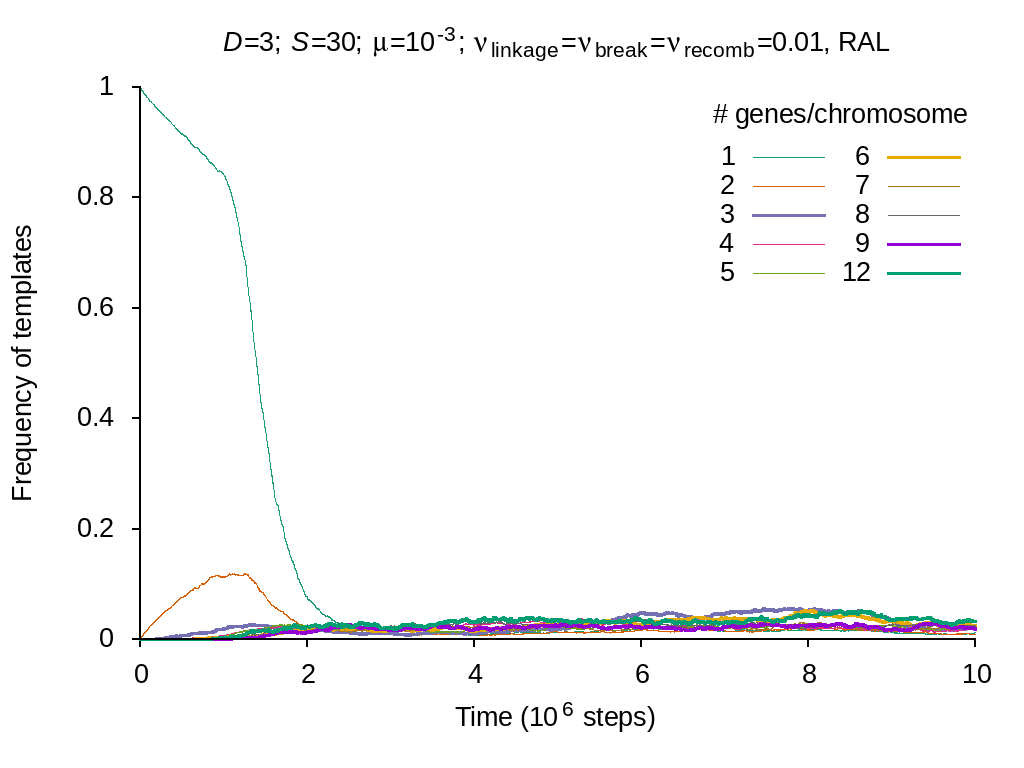  **S10 Fig. Frequency of different templates with RAL.** Frequencies are normalized on gene count, parameter values indicated at the top of the figure (standard parameter set as in Fig. 2) with reduced assortment load (RAL). Chromosomes consisting of 3·*n* (*n* positive integer) genes are plotted as thick lines.  On combining two treatments (RAL+DIRR) there are two opposite effects: RAL acts against chromosomatization while DIRR (probably) promotes it. With RAL+sDIRR chromosomes do not appear in any of the five investigated parameter sets. With RAL+lDIRR chromosomes appear after the transient period then disappear from the system, see S12 Fig. This can be understood as follows. Let us assume that an ABBCA chromosome breaks into two parts: ABB and CA. The replication rate of ABB is determined by the replication region of the gene in its head (A), which was the replication rate of the original chromosome. The replication rate of CA is determined by the replicative region of gene C. As there is no selective pressure for maintaining the replicative region of the non-head genes in a chromosome, continuous rounds of breakage-linkage would tend to equalize templates’ replication rates and, thus, would tend to minimize the within cell competition.  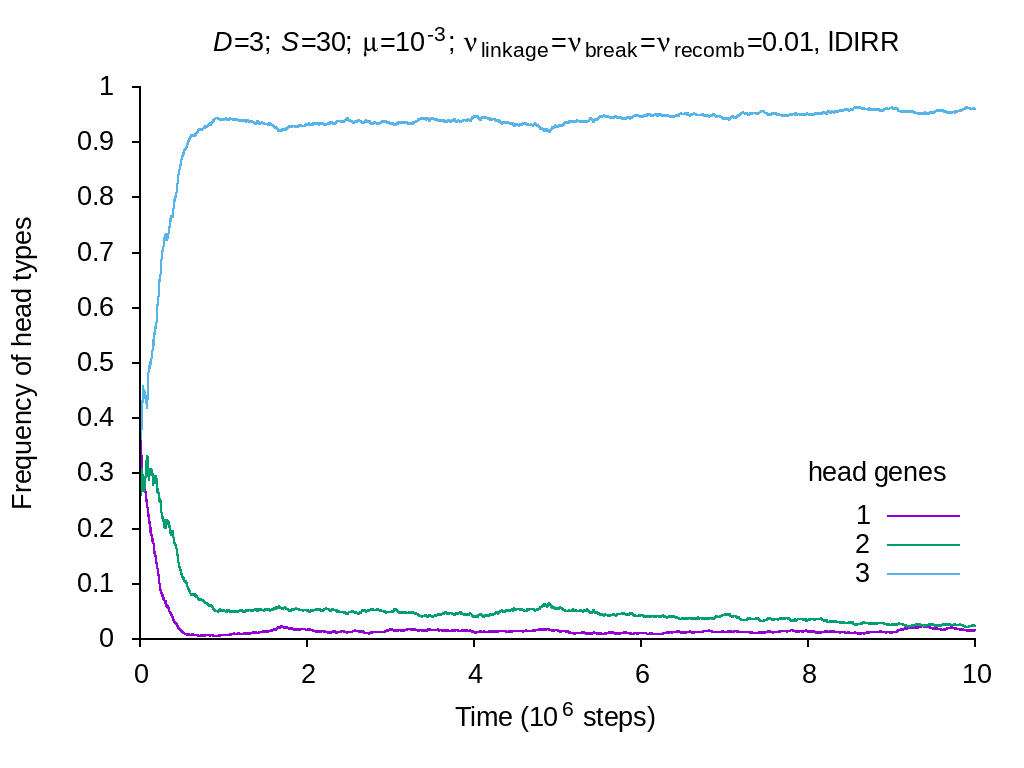  **S11 Fig.** **The frequency of head type in chromosomes (irrespective of the length of the chromosomes) with lDIRR.** Parameters are the standard parameter set as in Fig. 2.  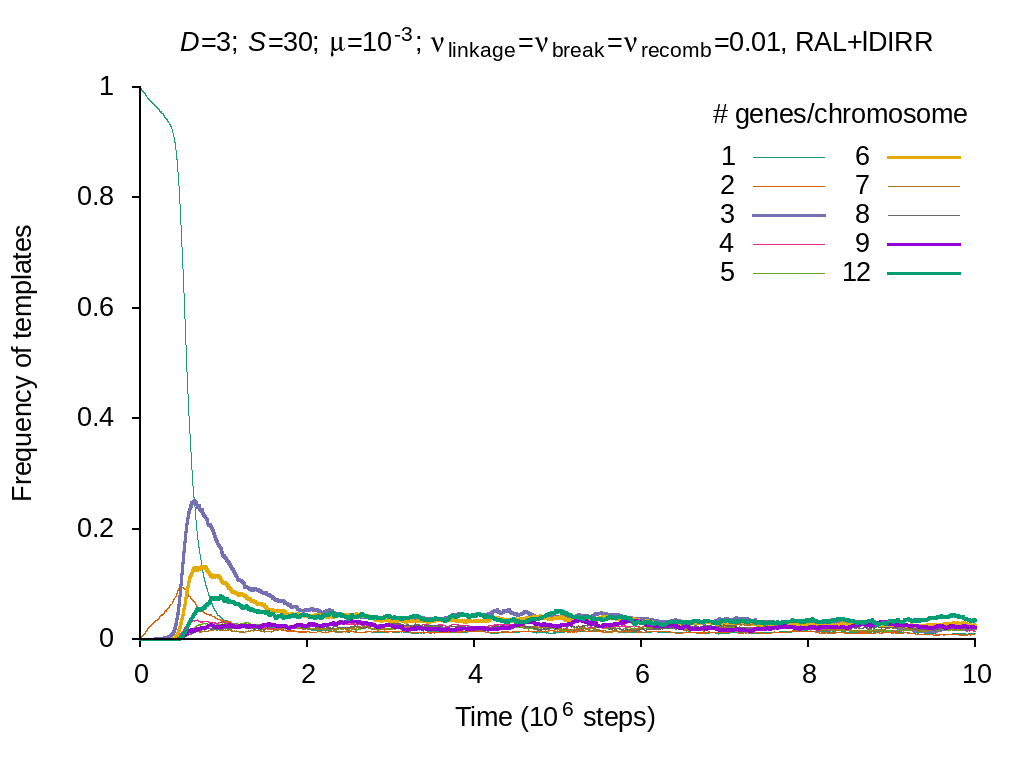  **S12 Fig. Frequency of different templates with RAL+lDIRR.** Frequencies are normalized on gene count, parameter values indicated at the top of the figure (standard parameter set as in Fig. 2) with reduced assortment load (RAL) and large differences in intrinsic replication rates (lDIRR). Chromosomes consisting of 3·*n* (*n* positive integer) genes are plotted as thick lines.   1. **Parameters of the model**   **S2 Table. Parameters of the model.**   \| **parameter** \| **description** \| **values** \| **ref.** \| \| --- \| --- \| --- \| --- \| \| *D* \| number of essential genes \| $2-8$ \| screen \| \| *η_t_* \| length of the target region for replicase \| 20 \| arbitrary \| \| *η_m_* \| length of metabolic region \| 80 \| arbitrary \| \| *S* \| split size \| $5-50$ \| screen \| \| α, β \| target affinity function parameters \| 5,15 \| [25] \| \| *µ* \| per bit mutation rate \| 0 – 8⋅10^-3^ \| screen \| \| $\nu_{\mathrm{link}},\nu_{\mathrm{break}},$  $\nu_{\mathrm{recomb}}$ \| probability of linkage, break and recombination \| 0, 0.01 \| screen \|   **S3 Table. Parameters of the figures.** The standard parameter set corresponds to Fig 2, the changed values marked by boldface.   \|  \| *D* \| *S* \| $\boldsymbol{\mu}$ \| $\boldsymbol{\nu}$ \| \| --- \| --- \| --- \| --- \| --- \| \| Fig 1 \| 3 \| 30 \| $10^{-3}$ \| **0** \| \| Fig 2 \| 3 \| 30 \| $10^{-3}$ \| 0.01 \| \| Fig 3 \| 3,**5** \| 30 \| $10^{-3}$ \| 0.01 \| \| Fig 4 \| $\boldsymbol{2-7}$ \| $\boldsymbol{5-50}$ \| $10^{-3}$ \| 0.01 \| \| S2Fig \| 3 \| **12** \| $10^{-3}$ \| 0.01 \| \| S3 Fig \| 3 \| **50** \| $10^{-3}$ \| 0.01 \| \| S4 Fig \| 3 \| 30 \| $\boldsymbol{6\cdot1}\mathbf{0}^{\mathbf{-3}}$ \| 0.01 \| \| S5 Fig \| **5** \| 30 \| $10^{-3}$ \| 0.01 \| \| S6 Fig \| 3,**5** \| 30 \| $10^{-3}$ \| 0.01 \| \| S8 Fig \| $\boldsymbol{2-7}$ \| $\boldsymbol{5-50}$ \| $\boldsymbol{0-8\cdot}\boldsymbol{1}\boldsymbol{0}^{\boldsymbol{-3}}$ \| 0.01 \| \| S9 Fig \| 3 \| 30 \| $10^{-3}$ \| 0.01 \| |
